# Subnational Analysis of Economic Fitness and Income Dynamic: The Case of Mexican States

**DOI:** 10.3390/e20110841

**Published:** 2018-11-02

**Authors:** Gonzalo Castañeda, Juan Romero-Padilla

**Affiliations:** 1 Centro de Investigación y Docencia Económica, Ciudad de México 01210, Mexico; 2 Colegio de Postgraduados, Estado de México 56230, Mexico

**Keywords:** economic fitness, complexity, regional development, growth forecasting, Mexican economy

## Abstract

In recent years, analytical tools of network theory have provided strong empirical support to the well-known hypothesis that regions develop through the local learning of capabilities (tacit productive knowledge). In this paper, we compare two indexes of competitiveness (or accumulated capabilities) for a subnational database of 32 Mexican states in the period 2004–2014. We find that Endogenous Fitness (i.e., region fitness and product complexity are derived jointly using only a Mexican exports database) has a better performance than Exogenous Fitness (i.e., product complexity comes from a world exports database and fitness is the sum of the complexity scores for the region’s competitive products). The performance criterion is established with the indicator’s capacity to meet a requirement of growth predictability: the existence of at least one *laminar (ordered) regime* in the fitness–income plane. In the Mexican data, Endogenous Fitness is a reliable predictor of per capita GDP in two distinct areas of the plane: one of continuous progress and opportunities, and another of stagnation and deteriorating fitness. The predictive capacity of this indicator becomes clear only when the metrics’ calculations are filtered by removing raw petroleum or oil-dependent states, while such capacity is robust to the inclusion of tourism—another important industry of the Mexican economy.

## 1. Introduction

Contrary to the propositions of conventional trade theory, the empirical evidence suggests that most countries are competitive in a variety of products. This evidence also indicates that the degree of diversification observed in the productive structure of countries is associated with their level of development [1,2,3]. In particular, richer countries tend to compete with many goods in international markets, some of them produced by very few countries and exhibiting a high added value, while the structure of poor countries is much less diversified and limited to unsophisticated goods facing many competitors [4]. 

Moreover, historical analyses of comparative economic systems assert that developing countries start a path of sustained growth after substantial modifications in their productive structure are undertaken, so that their industries are reconverted and their economies become more diversified [5,6,7,8,9,10]. Nonetheless, these structural transformations are not explained by neoclassical economists, nor are they thoroughly considered when formulating their growth theories (see Remark 1 in Appendix A1). However, these transformations and their implications on growth have been addressed by several scholars of alternative persuasions, as illustrated by pioneers of development economics [11,12,13], evolutionary economists [14,15], and post-Keynesian economists [16,17].

All in all, these studies argue that the productive diversity of a country (or region) is associated to its local capabilities; thus, economic development has to be conceived as a process where capabilities are created and adapted. In other words, growth is possible because the set of tacit productive knowledge widens through a dynamic of learning. These productive capabilities can be human (know-how), physical (infrastructure), and institutional (governance); whatever the case, they are a form of knowledge that is very difficult to transfer through patent acquisition, imitation, foreign direct investments, or imports. Accordingly, each region’s capabilities impinge critically on the nature of its productive structure and on the development path of its economy [18].

Recently, an innovative literature has emerged that endorses this point of view while providing a strong empirical support [19,20]. This line of research is a data-driven framework that uses network tools intensively. Likewise, it advocates a complexity vision that describes competitiveness as property emerging from a system with interactive productive units. Hence, economic development is seen as a form of exploration within a network, whose topology is described with a set of nodes that represent tradable products and links between nodes that are estimated through a measure of “proximity” [21,22] (see Remark 2). In this product space, any country’s productive structure (or export profile) can be characterized as the subset of nodes whose revealed comparative advantage (RCA) is relatively large (i.e., the country is competitive in producing such goods). 

Although this methodology was initially elaborated for analyzing the fitness (competitiveness or economic complexity) of countries, it is also possible to carry out subnational studies to determine the competitiveness of the regional economies of a particular country. This is so, regardless of the fact that within a country there is labor mobility and a relatively homogeneous institutional setting. The approach is valid at this level of aggregation if those capabilities that make one region more competitive than others are related to the superiority of its tacit knowledge, which is not easily transferred. Among these, organizational (e.g., logistics) and technological (e.g., trained workers) capabilities might be critical. Likewise, studying productive knowledge at a relatively disaggregated level is very important since it allows the identification of the geographical location of potentially competitive industries inside the national territory. It is precisely at the state level that a large set of industrial policies is commonly designed and implemented in reality. Therefore, our paper is framed in the very recent, but growing, literature of the subnational analyses of complexity and economic development [23,24,25,26,27,28,29,30,31].

Here, we calculate several indexes of fitness for the 32 states that compose the Republic of the United States of Mexico (the main reason for selecting the Mexican case is data availability; see Appendix A2 for details). Our main objective is to compare two alternative measures of fitness and evaluate them according to their potential to predict income growth. The first one, Endogenous Fitness, is computed directly from the framework’s metrics when applied to a database of products exported by Mexican states. The algorithm for calculating these metrics produces jointly indicators of product complexity and region fitness. The second one, Exogenous Fitness, is estimated indirectly through the arithmetic sum of the complexity scores that correspond to the competitive products in the state; previously, we have obtained such scores by applying these metrics to an international database of exports.

Once the rankings of these two indexes are computed and compared, we analyze the dynamic between per capita Gross Domestic Product (GDP_p_) and each of these two fitness indicators for the 32 states along the sampled period. This is done through a variant of a procedure known as “Selective Predictability Scheme” which does not impose ex-ante linearity between variables. The proposed variant is very helpful when the analyst has a very limited number of observations [28]. According to this method, a variable (fitness) has predictive capacity over another (GDP_p_) when there is a *laminar (ordered) regime* in a system defined in the fitness–income plane. That is, for any pair of coordinates in the area associated to the *ordered regime*, there is a high degree of predictability with respect to the direction that these variables will follow in the proximate future (around 10 years).

With these tools, we find empirical support to the hypothesis that Endogenous Fitness is more suitable for predicting purposes since the longitudinal data for the period 2004–2014 generates *laminar regimes* across the plane. Another interesting result is that there are two clear prospects for the Mexican regions. One for states with scant productive capabilities (either poor or heavily concentrated in few economic activities), where a daunting scenario prevails: a pattern of stagnation in GDP and regressive fitness. Another for states with a good backing of capabilities, where there is a virtuous circle: a pattern of continuous improvements in fitness along with positive growth in per capita income.

We divide the rest of the paper into six more sections and several appendixes. Section 2 explains how capabilities (an unobservable variable) can be inferred from a bipartite network established with the different states’ exported products (an observable variable). Section 3 specifies the mathematical formulas for the two metrics of fitness considered here and presents the associated rankings for the Mexican states. Section 4 describes the method of selective predictability and shows the empirical results for the dynamic of income and Exogenous Fitness. Section 5 explores the dynamics of income and Endogenous Fitness, once the sample is filtered by removing the most important export commodity in the country: raw petroleum. Section 6 evaluates the system predictability with an alternative indicator of competitiveness, which is frequently used in the literature: Economic Complexity. Section 7 conducts a sensibility analysis of the fitness index when a key service sector of the Mexican economy is included: tourism. Finally, the paper ends with a summary of the results and some additional considerations. 

## 2. Productive Capabilities and Regional Development

The growth of an economy is an evolutionary process where new products keep appearing in the region’s productive structure as new capabilities emerge [32]. According to empirical evidence, the new products tend to be more sophisticated than the established ones; likewise, some of the latter are displaced in a process of creative destruction, as suggested by Schumpeter [33] and estimated by [34] based on the complexity framework. Therefore, if we associate the level of economic development with the available productive knowledge, then it is imperative to measure the nature and quantity of these capabilities. If we could determine the existing capabilities in a region, it would be possible to specify with some degree of confidence the path of its economy in the near future.

Unfortunately, the capabilities located in different regions (or countries) are not easy to quantify directly, and it is even more difficult to make regional comparisons. This is the case because (1) we are dealing with a multidimensional concept whose elements are not clearly delineated and (2) there are no accepted standards to create comparable indicators nation-wise (or world-wise). The number of existing capabilities around the world is very large, especially if we realize that some of them are close substitutes (i.e., a social norm can provide the same functions as a formal rule). Even if the analysis only considers complementary forms of productive knowledge, it is impossible to establish a mapping from these capabilities to different outputs—as in a neoclassical production function.

Consequently, for a better understanding of how the productive structure of a region evolves, we need a methodology that estimates these capabilities indirectly from a set of observable and measurable variables. Because the goods sold through markets are the output of a process that articulates these capabilities, the production pattern of a region can be formally associated to its underlying capabilities. Thus, instead of working with a tripartite network (products, capabilities, and regions) that includes a non-observable variable (nodes of capabilities), it is preferable to work with a bipartite network (products and regions) based only on observable variables. 

The export profile of a region determines the location of its competitive nodes in the product space, and this, in turn, impinges upon the region’s development perspective (see Remark 3). Although all regions have the opportunity of gaining competitive advantages in new products while developing the required capabilities, the number and nature of such products are to a large extent conditioned by their current productive structures. In other words, each region’s subspace embodies its tacit productive knowledge and, hence, sets its growth potential. Thus, in order to infer the trajectory of income that a particular economy will follow in a time frame of 5–10 years, a first step is to establish a metric for evaluating the region’s degree of fitness (or sophistication) from the set of products that composes its export profile. 

With this aim, two groups of researchers [4,20] have built indexes of competitiveness that combine two features: how diversified an economy is and how ubiquitous their competitive products are. While the Latin American authors talk about the Economic Complexity Index (*ECI*), the Italian researchers make reference to the Fitness Index (*Fit*). These two features are related to the links of the bipartite network and reveal non-monetary information with regard to the abundance and sophistication of the economies’ embodied capabilities. That is, these researchers use alternative metrics of the nodes’ centrality in the network for measuring the complexity of regions and products.

Intuitively, a region that possesses a large variety of capabilities has a high probability of producing many goods (diversification), and if part of this knowledge is scantly shared with other regions, then some of its export will not have many competitors (ubiquity). Both features are essential for a good characterization of competitiveness. It is not enough to infer a high degree of complexity (fitness) for a region by observing that some of its exports have a reduced ubiquity, since this could only be a consequence of the availability of a very scarce natural resource worldwide (e.g., raw diamonds exported by Sierra Leona or Botswana). Additionally, one cannot infer the presence of a very sophisticated economy by noticing a relatively large amount of exported product (e.g., in manufacturing and agriculture), since this could only be the result of cheap labor and fertile conditions for arable land.

## 3. Two Forms of Measuring Economic Fitness

In this paper, we focus on the fitness index developed in [20,35]. These authors estimate jointly two types of indicators: one for the economic fitness of regions and another for the complexity of products. Their metrics are derived from the idea that products exported by developed regions provide relatively little information about the complexity of a product, because these regions tend to produce many things due to their vast productive knowledge. In contrast, developing regions compete in international markets with a short list of very simple products (e.g., commodities or low value-added goods). Therefore, although it make sense to measure the competitiveness of a region considering the summation of its competitive exports’ complexity, it is not convenient to measure product complexity as the average competitiveness of all the regions that export such a good (see Remark 4). 

Instead, a theoretically better definition of product complexity should, first of all, take into account that the sophistication of a good is negatively related with the number of regions exporting it (see Remark 5). That is, the more regions produce the good (high ubiquity), the lower its complexity tends to be. Secondly, this summation should give different weights to the regions producing it, so that highly diversified economies contribute very little to the sum. Hence, the fitness scores of all the regions that export the good competitively are added inversely. Therefore, a good that is produced by many regions with reduced fitness leads to its identification as a low complexity product. As can be inferred from this explanation, the metrics for the fitness of regions and the complexity of products are not associated linearly. Accordingly, they can be mathematically described by means of two coupled systems of non-linear difference equations:

(1) $${\tilde{F}}_{s}^{\left(n\right)}=\;\underset{P}{\sum }M_{sp}Q_{p}^{M\left(n-1\right)}\;$$

(2) $$\;{\tilde{Q}}_{p}^{M\left(n\right)}=\frac{1}{\sum_{s}M_{sp}\frac{1}{F_{s}^{\left(n-1\right)}}}\;$$

where $M_{sp}$ is a binary state-product matrix, so that $M_{sp}=1$ if the Mexican state “*s*” is competitive in exporting product “*p*” (i.e., if $RCA_{sp}\geq 1$) and $M_{sp}=0$ otherwise; $F_{s}$ is a vector that defines the fitness (competitiveness or sophistication) of each of the *S* states that integrate the dataset; $Q_{P}^{M}$ is a vector that indicates the complexity scores of the *P* products exported competitively by the Mexican states; *n* refers to the iteration number of a recursive process described by the difference equations (in the empirical analyses presented here, *n* = 100; see Remark 6); the tilde (~) on the endogenous variable specifies that the results obtained in each iteration are normalized by the mean value of the corresponding indicator; thus, $\;\;{\tilde{F}}_{s}$ y ${\tilde{Q}}_{p}^{M}$ are the indicators before normalization. The initial conditions for the algorithm are as follows: $F_{s}^{\left(0\right)}=1$ and $Q_{p}^{M\left(0\right)}=1$; however, after a large number of iterations, the coupled systems converge to the same values, independently of the initial conditions [35].

The coefficient of the revealed comparative advantage (RCA) for product “*p*” is then defined as the ratio between the share of exports of product “*p*” in the Mexican state “*s*” and the total exports of that state, divided by the share of exports of product “*p*” across states with respect to the sum of all Mexican exports. Mathematically,

(3)  RCAsp=x(s,p)∑px(s,p)∑sx(s,p)∑s,px(s,p). 

Consequently, the lower the fitness of a particular Mexican state ($F_{s}$), the higher the weight of that state in the summation, and the lower the complexity of the product. Moreover, it is important to notice in Equation (1) that, if a state becomes more diversified, its fitness score always increases. This does not necessarily happen with the averaging procedure of *ECI*, since the economic complexity of a region goes down when the productive structure is enlarged with a product whose complexity is lower than the average.

As an alternative to the metric of Endogenous Fitness (*EndoFit*), where the indicator is calculated using only information of Mexican exports, there is a variant that makes use of export data for a large set of countries. This variant is defined in [28] as Exogenous Fitness (*ExoFit*) since the scores for the products’ complexity are obtained through *EndoFit* applied to the world exports database. Then, these scores are used as weights in the summation of the states’ competitive products in order to establish the fitness indicator. Here, we define the RCA coefficient with the regional share of the product in the total of state exports divided by its international share in the total of world exports. The idea is that the complexity of a product is a notion related to the nature of the required capabilities (or tacit knowledge), independently of the region where it is produced. Hence, data from tradable goods at a world scale help to reduce the possibility of a bias in the indicator coming from the local conditions that prevail in a particular economy, such as trade barriers or subsidies. Consequently, the mathematical formulation for calculating this metric is as follows:

(4) $$\;\begin{array}{c} {\tilde{F}}_{s}=\;\underset{p}{\sum }M_{sp}Q_{p}\\ F_{s}=\;\frac{{\tilde{F}}_{s}}{{\tilde{F}}_{s}{}_{s}} \end{array}\;$$

where ${\tilde{F}}_{s}{}_{s}\;$ is the mean value across states of the non-normalized fitness indicator; $Q_{p}=\;Q_{p}^{M}=Q_{p}^{W}$ is the product complexity for the Mexican states’ export goods. This, in turn, is defined in terms of the product complexity calculated from the metric of Endogenous Fitness (Expressions 1 and 2) that uses the world exports database.

**Figure 1:**
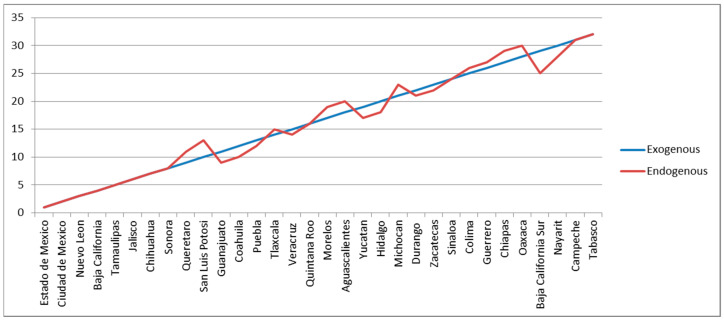
Rankings of Economic Fitness for the 32 Mexican states: 2014. The average of the yearly Spearman correlations between these two rankings for the period 2004–2014 is very high (${\bar{\rho }}_{ExEn}=0.9843$); however, the average of the yearly Cosine Similarity coefficients between their scores is markedly lower (${\bar{Cosine}}_{ExEn}$ = 0.8580).

A thorough analysis of the Mexican economic complexity at a subnational level is presented in [23,31]. In both papers, the *ECI* metrics are considered, but the former uses export data while the latter uses industrial employment data (i.e., agriculture is not included). In Figure 1, we show the rankings for the 32 Mexican states in 2014 calculated with the two alternative indexes of fitness. Despite the fact that *EndoFit* is measured using exclusively data of products exported by the Mexican states, these two rankings are extremely similar. Therefore, we may argue that both metrics produce similar classifications for the 32 states in terms of their economic fitness, and in this regard there is no “Mexican bias”. However, the reader should be aware that similar rankings do not imply that both indicators are equivalent in all respects and that they have the same predictive (or explicative) power. A high Spearman correlation does not necessarily mean that the magnitude of the differences across scores is preserved between indicators (see Remark 7 and Appendix A6).

Notice that in both rankings the State of Mexico is at the top followed by Mexico City; the former is a very industrious state surrounding the country’s capital. Many of the states with a high score of fitness are located on the Mexican border with United States (Nuevo Léon, Baja California, Tamaulipas, Chihuahua, Sonora, and Coahuila); some others in the north-central region of Mexico called “*El Bajío*” (Querétaro, San Luis Potosí, and Guanajuato), a dynamic and prosperous territory; plus Jalisco in the central-pacific region, whose capital is the second largest city in the country (Guadalajara). In the other extreme, some of the states with the lowest ranking in both metrics are located on the southern Mexican border or close to it (Guerrero, Chiapas, Oaxaca, Campeche, and Tabasco); the first three are relatively poor in terms of their per capita GDP, while the last two are oil-dependent economies. Two important exceptions with regard to the geographical position are Nayarit (central-pacific region) and Baja California Sur (northern-pacific region). Coincidentally, these two have relatively small economies with a heavy reliance on tourism, which is especially important in the latter state. 

## 4. The Dynamic of Income and Exogenous Fitness

In this section, we are interested in analyzing the dynamic process that relates a monetary indicator of aggregate performance (GDP_p_) with our intrinsic indicator of the productive knowledge available in the region (*ExoFit*). The purpose is to establish whether or not Economic Fitness can be a reliable measure to predict income growth in a span of 10 years. This can be done by means of a variant of a non-parametric prediction method called the Selective Predictability Scheme (SPS). This procedure was developed in [36] based on some features of the Method of Analogues, initially designed for weather forecasting [37]. The idea is that by discovering regularities in the pattern of time series, one can find situations in the past that are close to those currently observed and, from this, infer which will be the system trajectory in the near future. The SPS methodology was originally implemented for the analysis of the income dynamic at the country level (see Remark 8), but it was later modified for its application at a subnational level in [28], where the number of observations across states tends to be rather limited.

Before describing how this method operates, it is important to emphasize that it does not assume a linear relationship between GDP_p_ and Fitness or any other functional relationship. This is a crucial advantage since the traditional regression analyses used by economists assume parameter homogeneity across all observations. That is, econometric estimates describe the relationship between variables for a hypothetical unit that corresponds to the average observation value across regions and periods; hence, it is difficult to believe that the same estimated parameter holds for any unit (region-period) in the sample. Thus, in an attempt to circumvent this problem analysts estimate polynomial functions for specific independent variables (see Appendix A6). In contrast, in this non-parametric method, linear or non-linear relationships are possible, which means that the functional form has to be detected from the data and not imposed by the statistical model. Moreover, it takes into account that the non-linearity of a system not only has to do with the functional relationship between variables, but also with different dynamics that emerge depending on whether certain driving forces increase or decrease. 

Instead of using a measure of concentration that helps to delimit the predictability regions in the fitness–income plane, as in the original SPS, this newer version makes use of a measure of direction. The latter allows us to determine whether the vectors located in the threshold area of a specific cell of a grid, dividing the plane, tend to be positioned in parallel during the established time window [t_1_, t_2_]. In other words, the method estimates a coefficient ${\tilde{D}}_{k}\in \left(-1,\;1\right]$, which measures the dispersion of directions for all vectors that depart from the threshold area around the centroid of cell k in t_1_ and arrive at any point of the fitness–income plane in t_2_. We divide the plane with a grid of 100 × 100 cells whose extension is defined in terms of the data range, while the sides of the threshold areas are established with a specific bandwidth for each of the two axes. The smaller the value of ${\tilde{D}}_{k}$, the larger the dispersion of the vectors’ direction. This coefficient is calculated for a cell when at least three observations at t_1_ lie within the corresponding threshold area. For expositional reasons, the cells are colored according to the dispersion of observed trajectories in their threshold areas. A dark green color is used for cells whose vectors evolve in parallel, and a red color for cells whose vectors are directed unevenly. The mathematical expression for this measure of dispersion in the directions of motion is defined as an average dot product:

(5) $${\tilde{D}}_{k}=\frac{2}{N\left(N-1\right)}\overset{1,N}{\underset{i<j}{\sum }}{\hat{v}}_{i}\cdot {\hat{v}}_{j}\;$$

(6) with v^i= v→i|vi|;  v→i=aii^+bij^; |vi|=ai2+bi2; ai=log(Fi(t2))−log(Fi(t1))bi=log(GDPpi(t2))−log(GDPpi(t1)); i^=(1,0); j^=(0,1).

where *F_i_* is the fitness index; $\hat{i}$ and $\hat{j}$ are the versors (unit vectors) in the fitness and *GDP_p_* directions; *N* is the number of states whose starting coordinates lie inside the threshold area of cell *k*. Because the time range of the available data is limited, we select as a window [*t*_1_ = 2004, *t*_2_ = 2014]. 

Furthermore, we obtain estimates of the versors’ directions in regions of the plane where there is historic information by establishing another grid with wider cells (10 × 10). As performed in [28], we sum all vectors within each cell and then calculate its corresponding versor. In this fashion, we can detect empirically the type of regions that describe the evolution of Mexican states in the fitness–income plane. Theoretically, there can be a region with a “*laminar (ordered) regime*” where most versors (or arrows) present similar directions, but also a region with a “*chaotic regime”* where the arrows point toward different directions with no distinguishable pattern. When the initial coordinates of a state are located in a *laminar regime*, it can be argued that the fitness index has certain predictability with regard to the future evolution of GDP_p_, in contrast with the scenario that prevails when the state’s coordinates are positioned in a *chaotic regime* (see Remark 9).

Although per capita income and fitness are only two variables of a multidimensional vector that can help to predict economic growth, the methodology described above reduces this system to a bi-dimensional relationship when the *laminar regime* emerges. On the contrary, the *chaotic regime* can be empirically observed for two different reasons: (i) the system is indeed chaotic and, hence, similar initial conditions might exhibit very different trajectories, which ensues a low predictability; (ii) the multidimensional relationship cannot be synthetized with just two variables, so more information is required to understand the income dynamic in those states. In any case, if the data analysis shows the existence of different regimes, or several *laminar regimes* with different trajectories, this implies that the presumed non-linearity is, indeed, an empirical fact.

**Figure 2:**
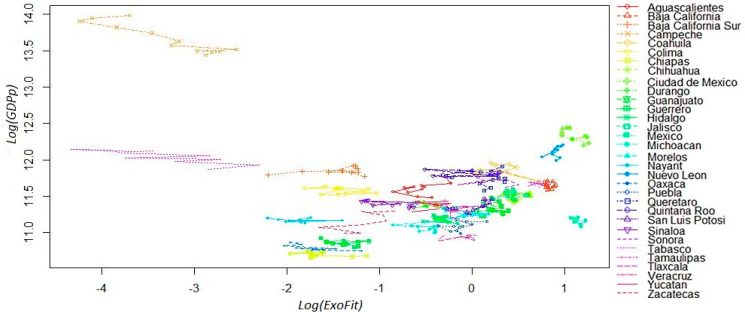
Fitness–GDP_p_ plane in logarithmic scale (yearly observations). *ExoFit* index for the 32 Mexican states: 2004–2014. Income in the vertical axis is defined as per capita GDP.

In Figure 2, we show the fitness–GDP_p_ plane in a logarithmic scale with the starting and ending coordinates for all yearly observations during the period 2004–2014 across the 32 states. In this case, we use the *ExoFit* metric for the horizontal axis. The most salient feature is that the trajectories of two particular states (Campeche and Tabasco) can be considered as outliers. The low fitness scores of such states seem to be in contradiction with their high GDP_p_. This paradoxical combination is the result of these economies being heavily dependent on crude oil production. This is a signal that, for Economic Fitness to be a good predictor of future income growth, it may be important to recalculate the indicator without oil production, or set aside those states that are very dependent on the exploitation of this natural resource. 

**Figure 3:**
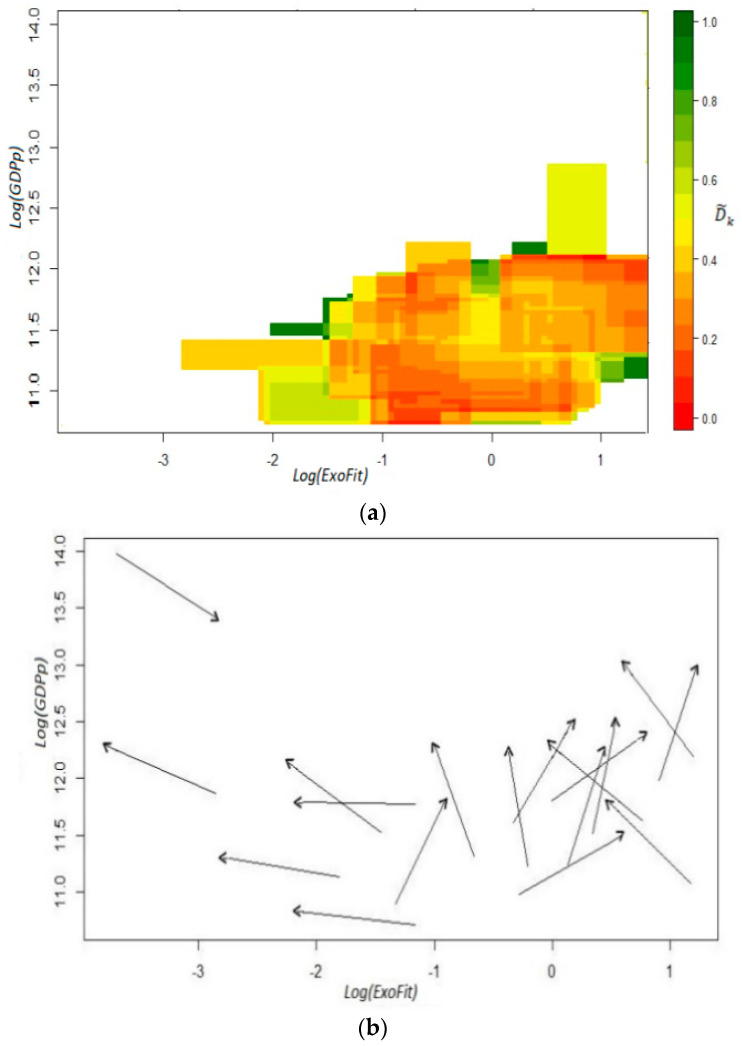
Fitness–income dynamic for the Mexican states. *ExoFit* index: *t*_1_ = 2004, *t*_2_ = 2014. Panel (**a**): coefficients ${\tilde{D}}_{k}$; panel (**b**): estimated versors. To obtain the coefficients ${\tilde{D}}_{k}$, we used an *x*-axis bandwidth of 0.86 and a *y*-axis bandwidth of 0.38. Income in the vertical axis is defined as per capita GDP.

Before the sample is filtered for a new calculation of the fitness scores, we show the coefficients ${\tilde{D}}_{k}$ in the top panel of Figure 3. As mentioned above, and for illustrative purposes, the cells whose estimated value indicates a high dispersion in the directions of motion are highlighted with the color red, while vectors that follow closely the same trajectory are distinguished with the color green. Notice that there are relatively few greenish cells in the grid and quite a few with a reddish tone; hence, the *ExoFit* indicator that includes the whole sample is a relatively poor predictor of growth, even for those states that are not oil-dependent (see Remark 10). This result is also evident in the bottom panel of Figure 3, where versors are estimated with wider cells. For states with a fitness value below −1 (in the logarithmic scale), there seems to be a *laminar regime* since most arrows tend to point toward a similar direction. However, this is a bizarre scenario since it indicates that states with a low Economic Fitness tend to grow slightly but at the expense of a narrowing productive structure. In contrast, for states with middle and high fitness, there seems to be a *chaotic regime* since arrows exhibit opposite directions.

## 5. The Dynamic of Income and Endogenous Fitness without Raw Petroleum

The odd trajectories that Tabasco and Campeche follow in the fitness–income plane indicate that one should be careful when applying the SPS methodology in countries where few regions export large amounts of a particular good. In fact, some analysts have pointed out that the fitness metric can be exceedingly sensitive to small variations in the network topology [38] (see Remark 11*)*. This is particularly troublesome in the case of a sparse bipartite trade network when there are niche products exported by a small number of regions. Accordingly, it is convenient to filter the sample and recalculate our indicators of competitiveness either discarding raw petroleum or excluding these two oil-dependent states. 

Firstly, we analyze the fitness–income dynamic by removing raw petroleum from the world exports database, and calculating Exogenous Fitness at the state level with the two-step procedure mentioned above. As can be seen in Appendix A3, the condition required for *ExoFit-oil* to be a good predictor does not hold since uneven directions seem to prevail in most cells across the plane. From these plots, it is not possible to infer that an improvement in a state’s productive structure leads necessarily to a sustained positive growth. Therefore, in this section, we evaluate the predictive capacity (reliability) of an endogenous index using only the Mexican exports database, where raw petroleum is also excluded from the metrics’ calculations (*EndoFit-oil*); likewise, we define income as per capita GDP without oil mining. 

**Figure 4:**
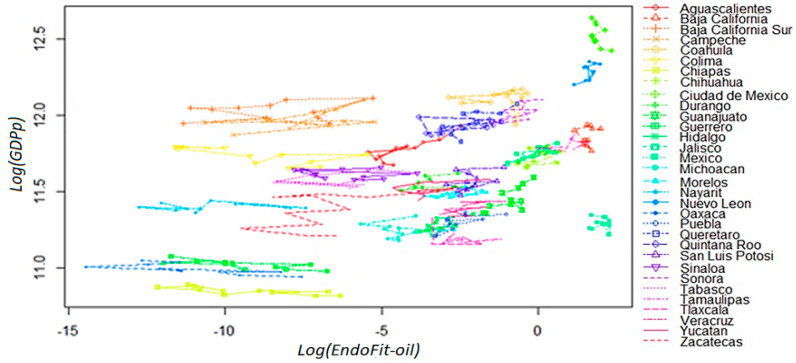
Fitness–GDP_p_ plane in logarithmic scale (yearly observations). *EndoFit-oil* index for the 32 Mexican states: 2004–2014. The Endogenous Fitness indicator was calculated without raw petroleum (product code = 2709). Income in the vertical axis is defined as per capita GDP without oil mining.

**Figure 5:**
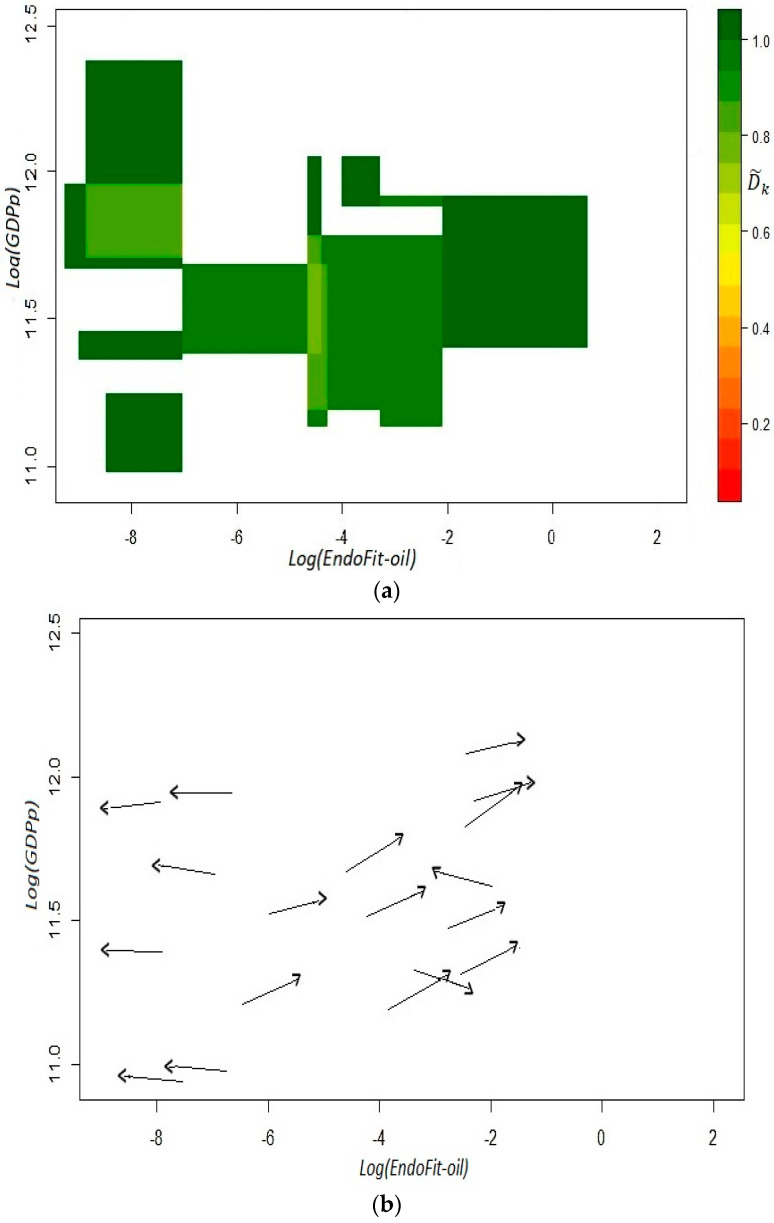
Fitness–income dynamic for the Mexican states. *EndoFit-oil* index: *t*_1_ = 2004, *t*_2_ = 2014. Panel (**a**): coefficients ${\tilde{D}}_{k};$ panel (**b**): estimated versors. The Endogenous Fitness indicator was calculated without raw petroleum (product code = 2709). To obtain the coefficients ${\tilde{D}}_{k}$, we used an *x*-axis bandwidth of 0.86 and a *y*-axis bandwidth of 0.38. Income in the vertical axis is defined as per capita GDP without oil mining.

As shown in Figure 4, in this setting, there are no isolated trajectories in the fitness–income plane, even for the oil-dependent states of Campeche and Tabasco. Moreover, there is a clear dynamic where there are neither high income trajectories for low fitness states, nor high fitness states that move through low income trajectories. Then, the estimated coefficients ${\tilde{D}}_{k}\;$ produce only green cells in the top panel of Figure 5, which is a remarkable result since it indicates high predictability for all the initial coordinates derived from the observations. However, instead of describing a large area with a homogeneous *laminar regime*, the versor drawn in the bottom panel signals the existence of two different areas with *ordered regimes*. 

For low fitness states, there is some sort of development trap since the trajectories for the six estimated arrows indicate a process of deterioration in productive capabilities as well as a stagnant economy. On the other hand, there is another laminar regime that predicts a promising future for states whose current *x*-axis coordinate in the plane corresponds to scores of middle and high fitness. In this regime, states will not only exhibit a positive growth but also a continuous sophistication of its productive structure. These two results combined suggest that unproductive states (poor or oil-dependent) require the building up of capabilities for some years in order for their economy to enter into a virtuous cycle of growth and opportunities (see Remark 12). From a technical point of view, the presence of different ordered regimes is an empirical corroboration that the linear analysis of growth regressions is faulty and can result in misguided advice, as in [18].

**Figure 6:**
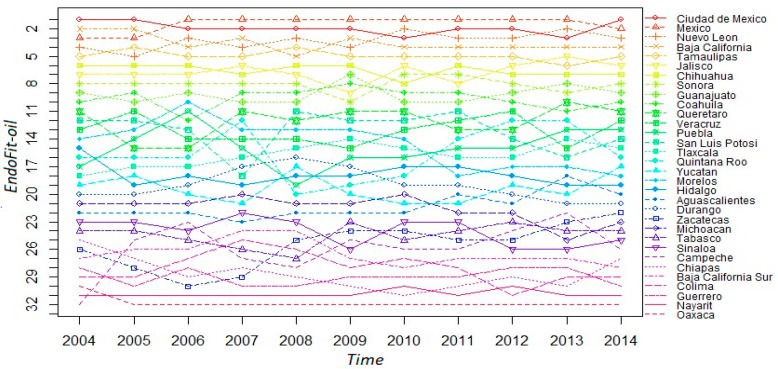
Ranking evolution of the Mexican states’ fitness. *EndoFit-oil*: 2004–2014.

Once we correct for oil-dependency, the *EndoFit* index meets our requirement of predictability; hence, we can assert that this metric can be a reliable indicator for forecasting the evolution of income, at the subnational level, in the Mexican economy. We present some sensibility tests in Appendix A4, where we recalculate the competitiveness indexes by removing the oil-dependent states from the sample. These exercises indicate that Endogenous Fitness is a robust indicator, at least in providing proper conditions for the predictability of economic growth. Therefore, we proceed in Figure 6 to describe the ranking for the 32 states according to this index. Notice that there are no radical changes across the sampled years; inclusively, the ranking is rather stable for the top 8 states. Likewise, the bottom 9 states for 2014 are the same as those presented in the list of Figure 1, where petroleum is included in the analysis. However, there are some changes in positions, with the case of Tabasco being the most notorious. This state moves from the last position to 24th place in the alternative ranking, while the state of Campeche also moves up in the ladder. This result implies that the fitness index with the complete sample penalizes those states whose economy depends heavily on the exports of one product. Furthermore, when the aim is to infer the growth of aggregate income without oil, it seems that excluding such a product from the fitness calculation is an appropriate advice.

## 6. The Dynamic of Economic Complexity and Income 

In this section, we present an additional analysis to assess whether the competitiveness indicator based upon product complexity derived from the metrics of Economic Complexity (i.e., the Latin American variant) surpasses our predictability requirement. This is also an exogenous index since the *ECI* scores for the Mexican states come from a weighted sum of their competitive products in international markets, where the weights are defined in terms of the product’s complexity calculated with a panel of countries. The states’ scores for this indicator come from *El Atlas de la Complejidad Económica de México* (*f*or a description of the Mexican exports database, see Appendix A2). 

When we compute the average of the yearly Spearman correlations for the two exogenous indicators (*ExoFit* and *ECI*), we notice that the estimate is relatively high: ${\bar{\rho }}_{ExEci}=0.91089$. However, as mentioned above, this result does not mean that both indexes are identical in all dimensions or that similar implications will be produced with any of these indicators. It is important to have in mind that this correlation coefficient is only one possible measure of similitude; thus, this finding does not guarantee that both indexes offer equivalent conditions for predictability. In fact, when the average of the yearly Cosine Similarity coefficients is estimated, the degree of similitude falls by 0.14289 (${\bar{Cosine}}_{ExECI}=\;0.7680)$.

**Figure 7:**
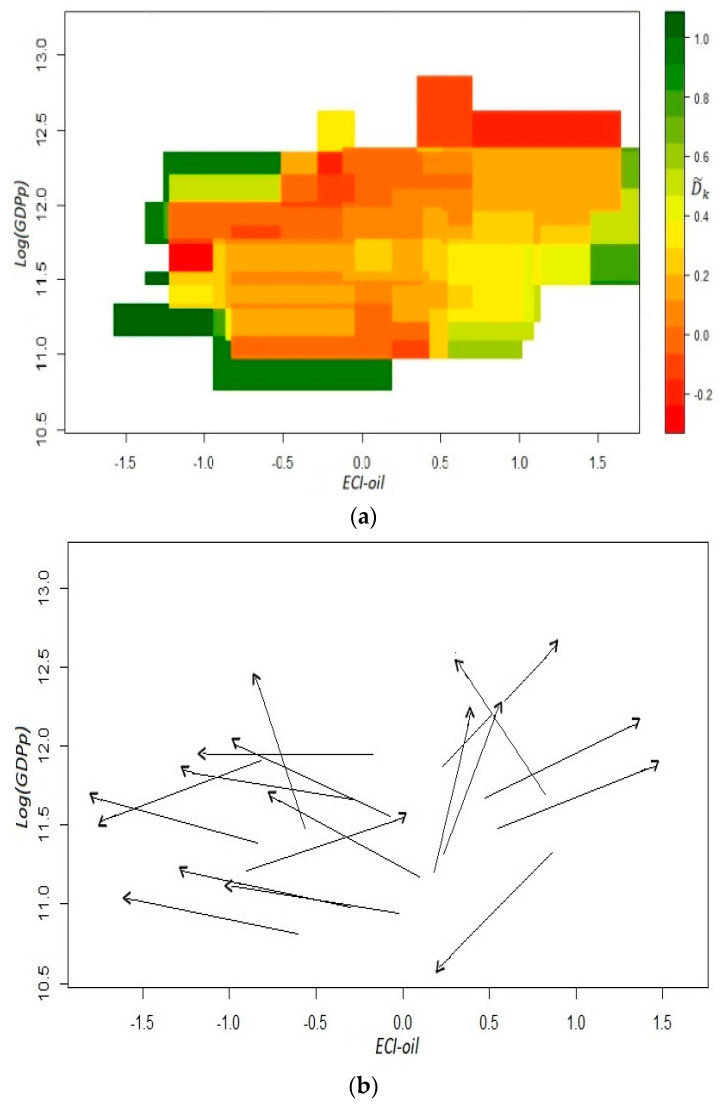
Fitness–income dynamic for the Mexican states, *ECI-oil*: *t*_1_ = 2004, *t*_2_ = 2014. Panel (**a**): coefficients ${\tilde{D}}_{k}$; panel (**b**): estimated versors. To obtain the coefficients ${\tilde{D}}_{k}$, we used an x-axis bandwidth of 0.86 and a y-axis bandwidth of 0.38. Income in the vertical axis is defined as per capita GDP without oil mining.

Anew, our dynamic analysis is based on the coefficients ${\tilde{D}}_{k}$ and the estimated versors in the fitness–income plane. The measure that we use for aggregate income comes, again, from per capita GDP excluding oil mining. From both panels of Figure 7, it is more than evident that our predictability requirement is not met. As in Figure 3, there is a significant dispersion of directions in most of the cells that can be empirically analyzed with the data. Moreover, there seems to be a non-linear relationship between *ECI-oil* and income since for states with an indicator below a certain level of competitiveness, *ECI-oil* tends to go down, while the opposite occurs for states with a relatively high level of productive knowledge. However, the income–fitness dynamic is not as straightforward as the one observed in the previous section with the *EndoFit-oil* index. A similar outcome is shown in Appendix A4 when *ECI* is evaluated removing the two oil-dependent states.

## 7. How Sensitive is the Fitness Index to the Inclusion of Tourism?

The results presented in sections four and five indicate that a very important product for a country and, in particular, for some of their states might interfere with the potential of the Selective Predictability Scheme as a forecasting tool. Consequently, in this section we analyze the effects of another key industry for some of the Mexican states: tourism. Because this is a service sector and the methodology for quantifying regions’ competitiveness is based mainly on exported goods, we make some arrangements in our database to include the value of tourism for each state in the metrics’ calculations. Due to data limitations, this is done here by estimating the income spent by foreign nationals when visiting a Mexican state (see Appendix A2 for details). 

**Figure 8:**
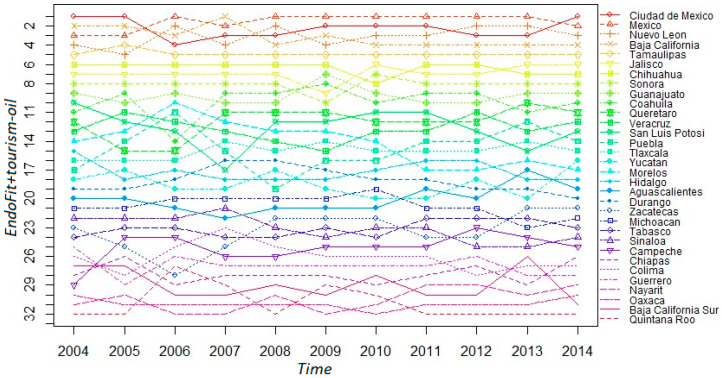
Ranking evolution of the Mexican states’ fitness. *EndoFit + tourism-oil:* 2004–2014.

We recalculate Endogenous Fitness excluding raw petroleum and including tourism as another item of the productive structure (*EndoFit + tourism-oil*). Then, we present in Figure 8 the evolution of states’ rankings using this metric. In general, the rankings are not very different from those shown in Figure 6 where the indicator of Endogenous Fitness is only corrected by excluding raw petroleum. The stability of the rankings is also clear-cut; the top 12 positions are identical; 12 out of the 13 bottom states are the same, although in different positions. However, the state of Quintana Roo—where the touristic resorts of Cancun and Riviera Maya are located—moves drastically in the ranking; from 16th position to last position. Baja California Sur—where the resort of Cabo San Lucas is located—also goes down in the ranking, but to a much lesser extent; from 28th position to 31st. 

**Figure 9:**
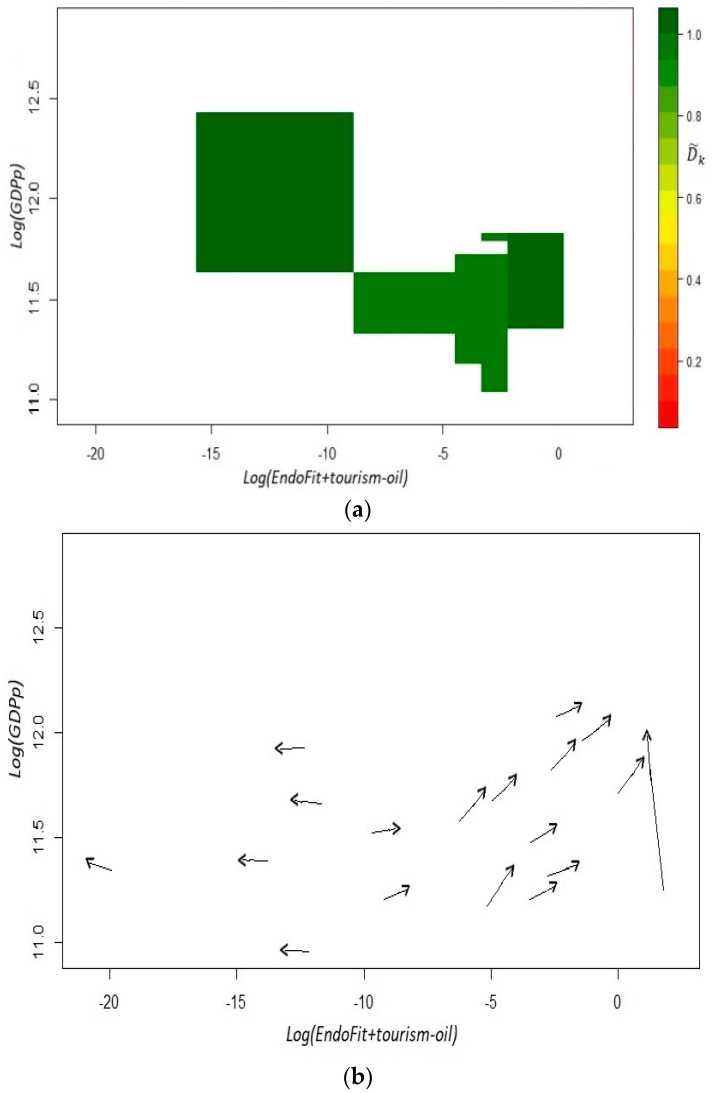
Fitness–income dynamic for the Mexican states, *EndoFit+tourism-oil*: *t*_1_ = 2004, *t*_2_ = 2014. Panel (**a**): coefficients ${\tilde{D}}_{k}$; panel (**b**): estimated versors. Income is defined as per capita GDP without oil mining. To obtain the coefficients ${\tilde{D}}_{k}$, we used an *x*-axis bandwidth of 0.86 and a *y*-axis bandwidth of 0.38. Income in the vertical axis is defined as per capita GDP without oil mining.

With this result, we can conclude, again, that including industries that are extremely important for a region has negative consequences for its competitiveness score. Theoretically, this feature can be deleterious for the economy’s growth, if the concentration of the economic activity generates a more volatile environment or deters the creation of new capabilities through some form of “Dutch disease’. Here, we only want to determine empirically, if our index of *EndoFit* corrected by tourism loses or improves its predictive capacity. As can be seen from both panels of Figure 9, the dynamic of the system is still predictable. Even more, we can say that the estimated coefficients ${\tilde{D}}_{k}\;$ have a more uniform color of dark green in the different cells, although not in an ostensibly manner. Likewise, the plot of estimated versors in the fitness–income plane still identifies two *laminar regimes*. This outcome reaffirms the finding that in the Mexican economy there are two dynamic settings: a development trap for unproductive and low fitness states and a virtuous cycle for relatively rich and high fitness states. 

Finally, in Appendix A5, we modify the specification of RCA by dividing the export value of a product in a state and in Mexico by population instead of the total export value in the corresponding region. This approach produces coefficients that are neutral to price changes, and precludes that the measurement of the products’ competitiveness, in a particular region, can be overshadowed by an industry with an extremely high export value. However, we find with our database that the indicator of Endogenous Fitness does not qualitatively improve its performance with this adjustment.

## 8. Conclusions

The conception of economic development as a decentralized process of exploration where productive capabilities change and accumulate through time has been stimulated in recent years. The tools of network theory and the complexity vision of economic systems offer explanations for the competitiveness of nations and provide tests for empirical validation. Furthermore, this perspective contributes in designing innovative frameworks for public policy and forecasting. In particular, several indicators have been built and implemented to measure a region’s ability to compete as a result of the productive knowledge embodied in its economy. According to the literature, these non-monetary indicators convey information that is very relevant for inferring the development potential of a region; thus, they are a valuable metric for forecasting economic growth.

In general, for these tools and frameworks to be considered methodologically robust, it is important to analyze their performance with different databases. Therefore, in this paper, we study whether or not the condition required for the predictability of growth holds when using an indicator of fitness (competitiveness) with a subnational database of the 32 Mexican states. This requirement consists in observing an extended area of the fitness–income plane characterized by a laminar regime; that is, by a set of initial coordinates where the analyst can infer the trajectories that per capita GDP can follow in a range of 10 years. Our results show that the endogenous variant of this fitness indicator has a better performance than the exogenous one; in the former indicator “state fitness” and “product complexity” are calculated jointly from the Mexican exports database, while in the latter “product complexity” is derived from a world exports database. Moreover, our empirical analysis points out that the condition for a reliable forecast is met once the indicator is corrected by removing raw petroleum from the sampled products—a very important export commodity for Mexico and, especially, for the states of Campeche and Tabasco (see Remark 13).

Likewise, the application of the SPS for the Mexican case suggests the presence of two Mexicos: a productive one in the north and central parts of the country and an unproductive one in the South. Hence, several states are situated in a development trap of stagnation and a deteriorating productive structure. This result is somehow robust since it is observed in most of the fitness indicators considered here. Consequently, for a poor state to observe a surge in its per capita GDP, it requires a deep structural transformation. Marginal increases in fitness, and the slow building up of capabilities, do not guarantee a rapid take off of the region. Hence, an industrial policy is required for a large jump in productive knowledge to be possible and for the economy to move into a path of sustained growth. In other words, for these states to enter into a virtuous circle of growth and productive opportunities, a comprehensive industrial policy needs to be implemented with the aim of creating an environment conducive to the development and articulation of new capabilities.

From the complexity perspective, these policies should not be entirely developed from the top-down. Instead, their conception and implementation should be part of an ecosystem that encourages public, social and private agents to provide ideas, funds, and the processing of information. An environment that fosters the interaction of different insights enables a boost in aggregate productivity and an inclusive process of economic development. The policy menu in this ecosystem can be very wide: (i) the establishment of autonomous and decentralized councils of industrial coordination at the state/municipal level, to be in charge of managing socially oriented venture capital funds (see Remark 14); (ii) ex-ante guarantees in industries that are new for the region, which do not have to be paid *ex-post* when these projects happen to be successful (see Remark 15); (iii) a system of contests where projects of small and medium firms are awarded with funding due to their inventiveness and the strategic value of the proposed investments in the community (see Remark 16); (iv) the creation of critical infrastructure conducive to the crowding-in of private investments, such as roads, environmentally friendly sources of energy, train terminals, and industrial parks with free trade benefits (i.e., with their own customs and the avoidance of revisions in the ports of entry); (v) the establishment of “special economic zones” in populated but very depressed regions that require strong fiscal incentives to encourage very large investments, especially those that are labor-intensive [39,40]; (vi) the creation of information agencies at the federal level with a mandate to provide historical and updated data on economic activity, exports–imports, employment, and occupations at municipal and state levels, so that consultants, local governments, academicians, and entrepreneurs can produce meaningful analyses of regional development (see Remark 17). 

It is important to emphasize that the results presented in this paper highlight the fact that analysts and practitioners should not use these metrics mechanically. The type of indicator considered (exogenous versus endogenous) and the way the regions and products are filtered can produce contrasting implications. As our exercises of excluding (including) raw petroleum (tourism) in the fitness indicators show, there is no assurance that a specific set of procedures will work in any context. For the time being, the intrinsic instability of the fitness algorithm (i.e., the coupled systems of non-linear difference equations) has to be balanced with a variety of sensibility analyses. Hopefully, in the near future, more reliable metrics will be developed so that a set of network tools can be implemented following standard protocols, such as those currently used in econometric analyses. 

Lastly, it is important to recall that a forecasting technique only indicates which outcomes are likely to be produced in the future when the environment remains unperturbed. Therefore, knowing that a region is positioned in a development trap is only a first step in recognizing that a new environment is required for the economy entering into a process of sustained development. New tools have to be elaborated to study how alternative settings can produce a substantial improvement in the economy’s medium-term performance. For instance, it is important to know the proper policy priorities for a region to attain the multiple targets specified by a particular development mode [41]. Moreover, it is clear that it will be impossible for a laggard region to start being competitive in industries with a large strategic value when certain socioeconomic indicators have not achieved the required level (e.g., some of these industries can be highly dependent on the existence of a well-functioning public governance) [42]. 

## Figures and Tables

**Figure A1:**
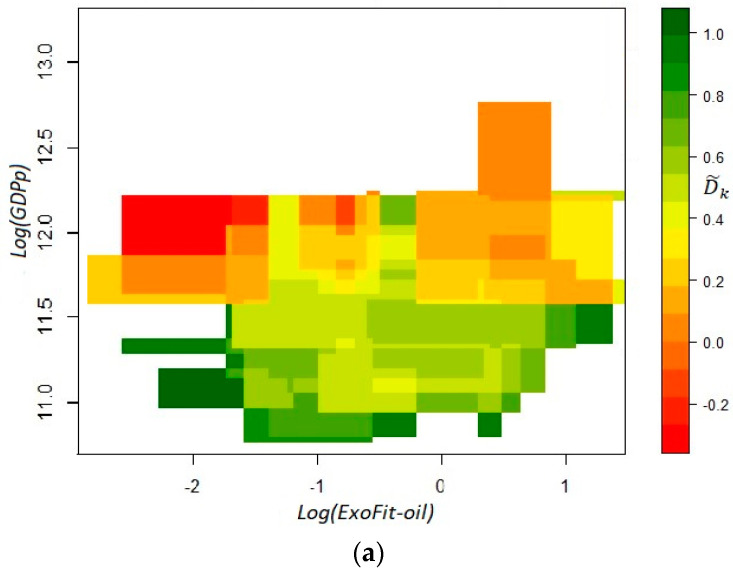

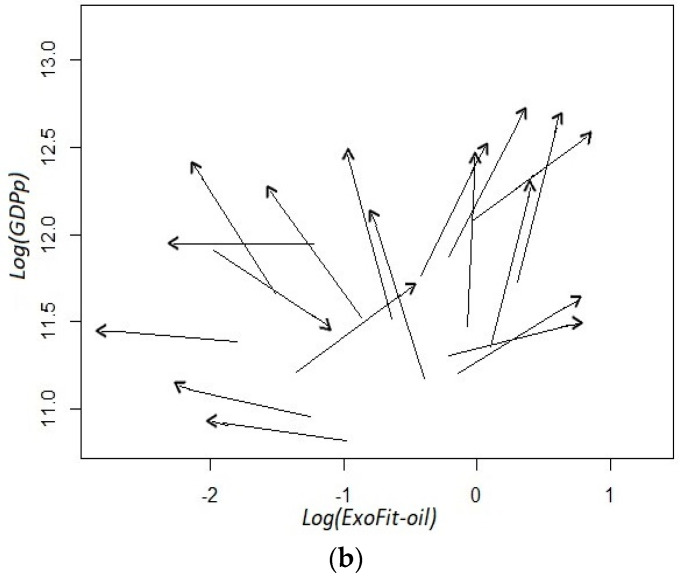
Exogenous Fitness without raw petroleum. *ExoFit-oil*: *t*_1_ = 2004, *t*_2_ = 2014. Panel (**a**): coefficients ${\tilde{D}}_{k}$; panel (**b**): estimated versors. To obtain the coefficients ${\tilde{D}}_{k}$, we used an x-axis bandwidth of 0.86 and a y-axis bandwidth of 0.38. Income in the vertical axis is defined as per capita GDP without oil mining.

**Figure A2:**
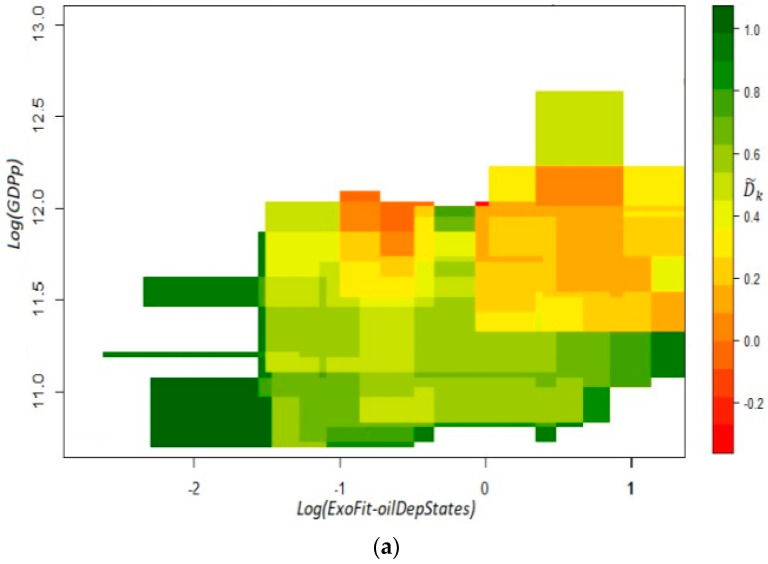

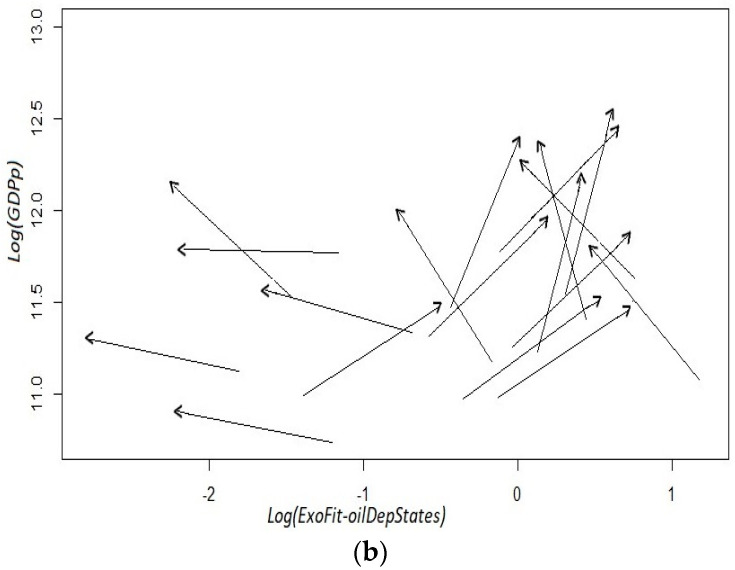
Exogenous Fitness (*ExoFit*) without oil-dependent states: *t*_1_ = 2004, *t*_2_ = 2014. Panel (**a**): coefficients ${\tilde{D}}_{k}$; panel (**b**): estimated versors. To obtain the coefficients ${\tilde{D}}_{k}$, we used an *x*-axis bandwidth of 0.86 and a *y*-axis bandwidth of 0.38. Income in the vertical axis is defined as per capita GDP.

**Figure A3:**
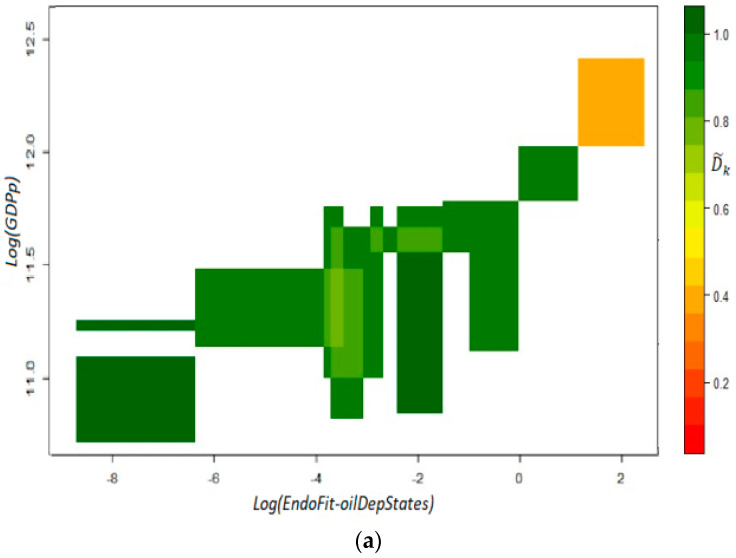

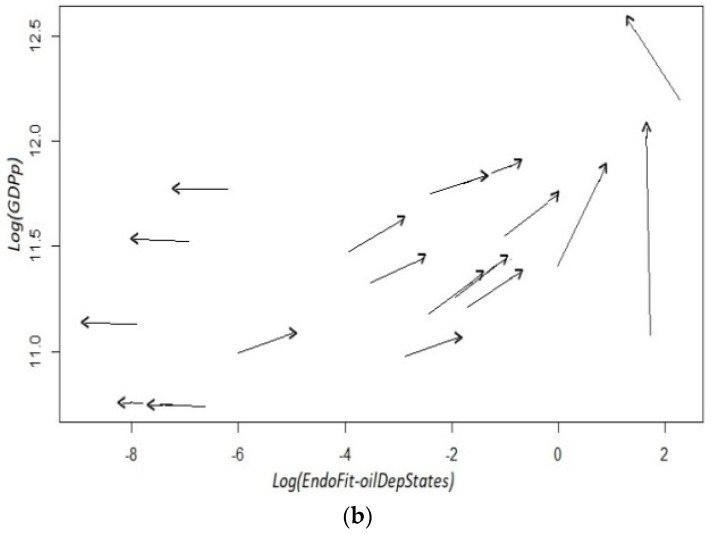
Endogenous Fitness (EndoFit) without oil-dependent states: *t*_1_ = 2004, *t*_2_ = 2014. Panel (**a**): coefficients ${\tilde{D}}_{k}$; panel (**b**): estimated versors. To obtain the coefficients ${\tilde{D}}_{k}$, we used an *x*-axis bandwidth of 0.86 and a *y*-axis bandwidth of 0.38. Income in the vertical axis is defined as per capita GDP.

**Figure A4:**
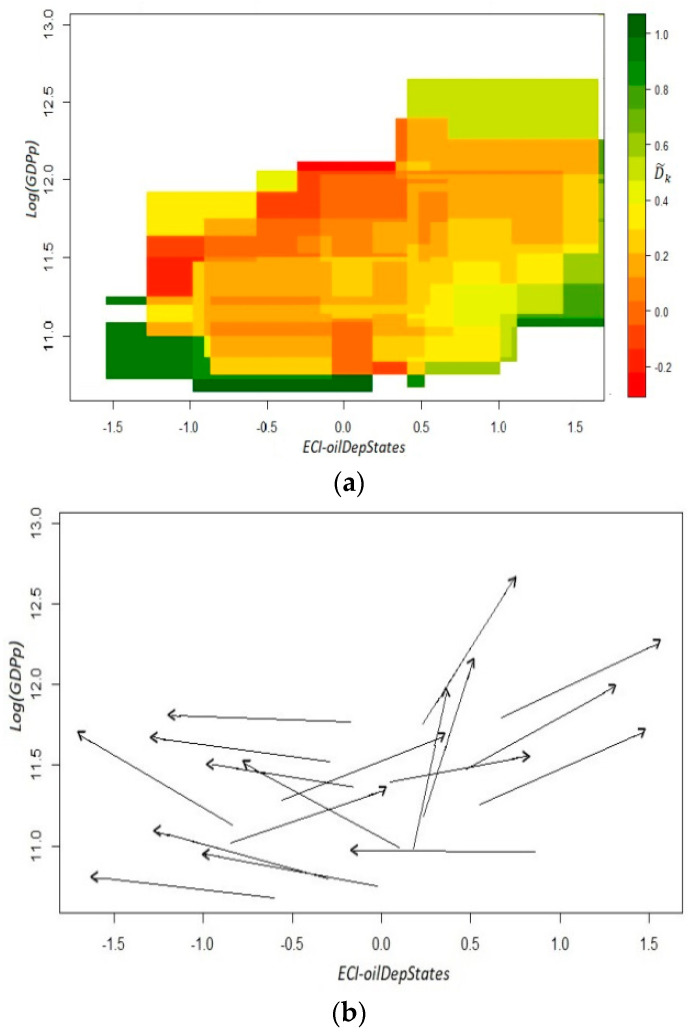
Economic Complexity (ECI) without oil-dependent states: *t*_1_ = 2004, *t*_2_ = 2014. Panel (**a**): coefficients ${\tilde{D}}_{k}$; panel (**b**): estimated versors. To obtain the coefficients ${\tilde{D}}_{k}$, we used an *x*-axis bandwidth of 0.86 and a *y*-axis bandwidth of 0.38. Income in the vertical axis is defined as per capita GDP.

**Figure A5:**
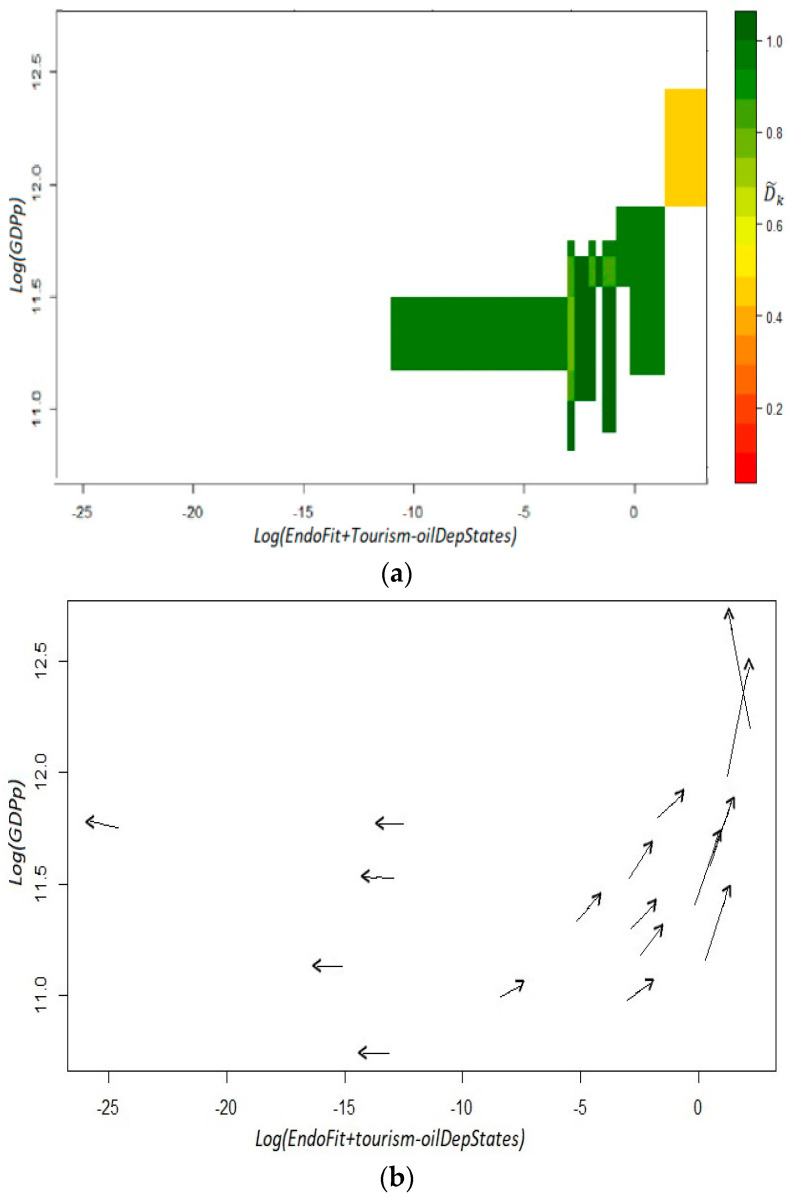
Endogenous Fitness with Tourism (*EndoFit+Tourism*) without oil-dependent states: *t*_1_ = 2004, *t*_2_ = 2014. Panel (**a**): coefficients ${\tilde{D}}_{k}$; panel (**b**): estimated versors. To obtain the coefficients ${\tilde{D}}_{k}$, we used an *x*-axis bandwidth of 0.86 and a *y*-axis bandwidth of 0.38. Income in the vertical axis is defined as per capita GDP.

**Figure A6:**
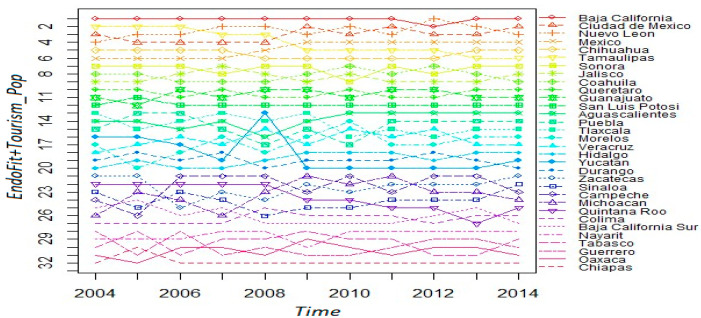
Ranking evolution of the Mexican states’ fitness, *EndoFit~Pop*: 2004–2014.

**Figure A7:**
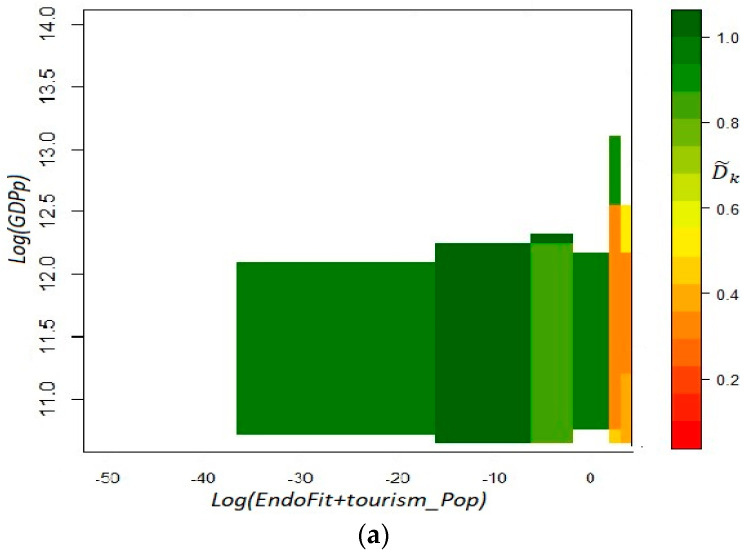

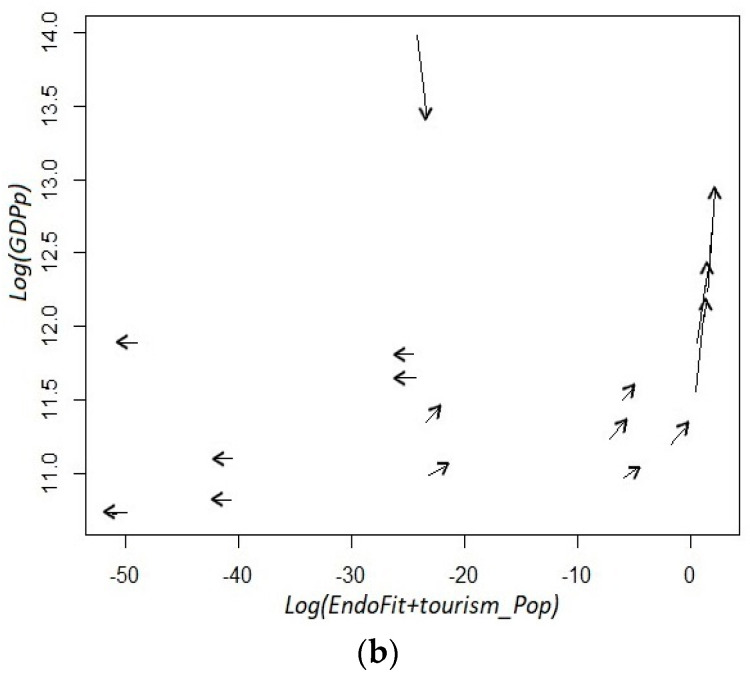
Endogenous Fitness with Tourism normalized by population (*EndoFit+Tourism~Pop*): *t*_1_ = 2004, *t*_2_ = 2014. Panel (**a**): coefficients ${\tilde{D}}_{k}$; panel (**b**): estimated versors. To obtain the coefficients ${\tilde{D}}_{k}$, we used an *x*-axis bandwidth of 1.3 and a *y*-axis bandwidth of 1.0. Income in the vertical axis is defined as per capita GDP.

**Figure A8:**
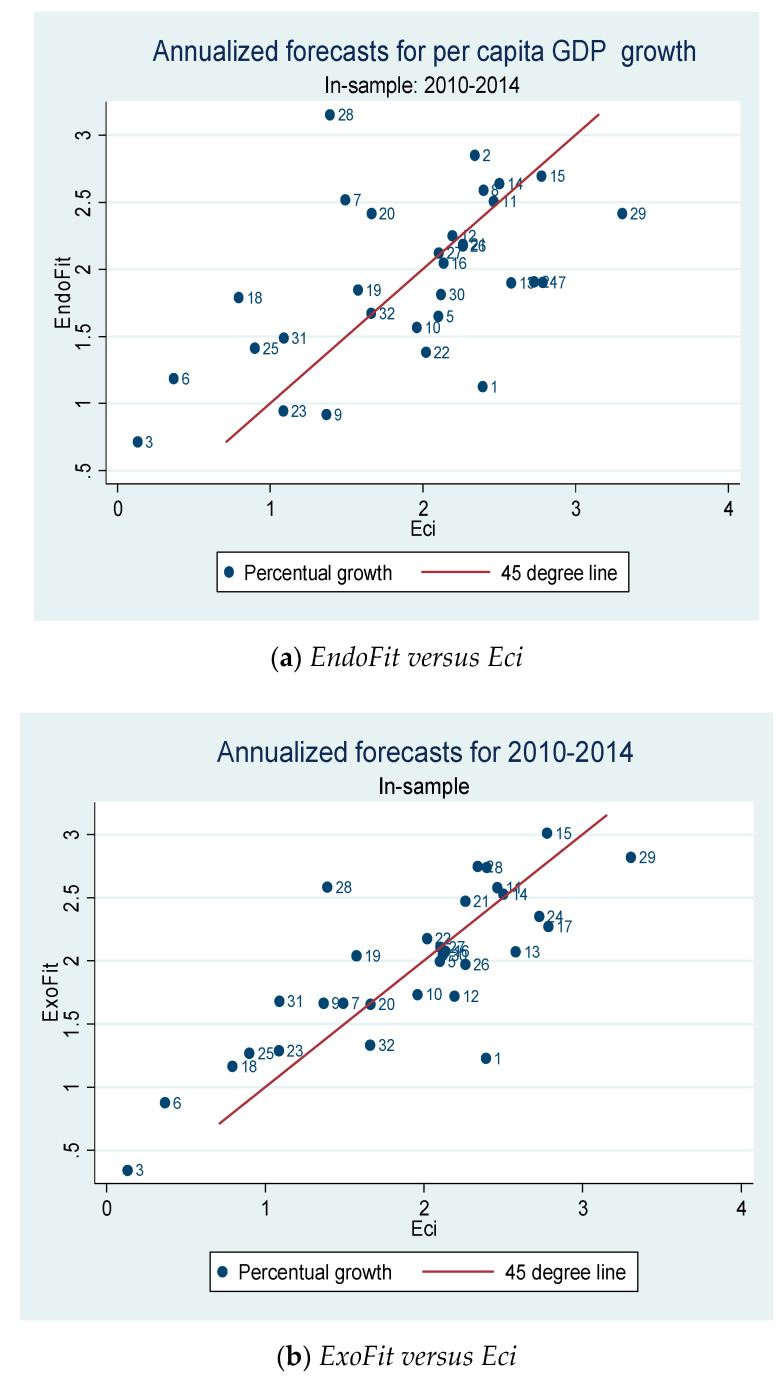
Growth predictions with random effects estimations: These graphs are drawn with the adjusted values obtained from regressions of Table A5; only predicted income growth for 2010–2014 is presented (annualized); income growth is defined with per capita GDP, inflation adjusted; predicted values come from using xb; the point estimates for Campeche are excluded for improving the clarity of the illustrations (this is the only state with a negative growth in the sample).

**Figure A9:**
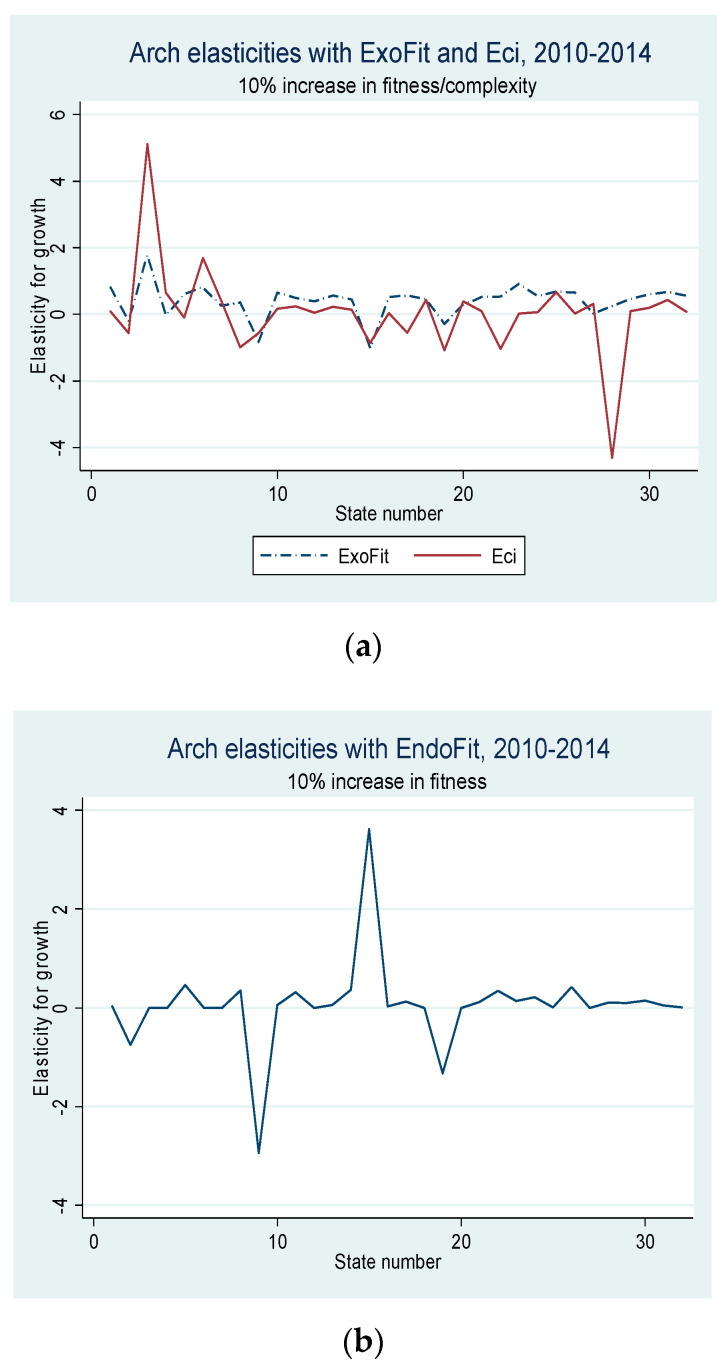
Arch elasticities for growth calculated from the random effect estimations. These graphs are drawn with the adjusted values obtained from regressions of Table A5; only arch elasticities for predicted growth in the period 2010–2014 are presented, these are calculated with a 10% increase in the fitness/complexity indicator; income growth is defined with per capita GDP, inflation adjusted; predicted values come from using xb.

## Appendix A

### Appendix A1. Remarks

**Remark** **1.**
*In fact, there are some endogenous growth models that analyze structural transformations. However, these models postulate production functions a la Dixit–Stiglitz that assume a continuum of goods with the same cost function as well as constant and identical elasticities of substitution for any pair of goods. In this type of models, technology is transformed endogenously through some process of positive feedback (e.g., learning, externalities, and increasing returns) between the scale of inputs and productivity [43,44,45,46]. Therefore, they assume an extremely simplified productive structure, in so far as they do not take into account the wide heterogeneity observed in traded goods. Because the economic setting of these models is rather unrealistic, they are not very helpful for producing empirical analyses of the evolutionary patterns observed in the countries’ productive structure.*

**Remark** **2.**
*It is said that two products are rather similar when they share the same capabilities; thus, their corresponding nodes have a link whose weight is given by the ratio of the number of countries that export it jointly with respect to the maximum number of countries that export any of them. Moreover, in [47], a taxonomy network is built where a directed link goes from node a to node b if the production of a makes the future production of b more likely. This hierarchical network helps to identify which development paths in the product space are easier to undertake for a particular country.*

**Remark** **3.**
*Ideally, for the estimations of competitiveness and the product space, we would like to consider all sets of goods and services produced in each region. However, there are no disaggregated data available for non-tradable goods or services, whether the latter are sold through international markets or internally. In any case, in [18,36], the authors show that indicators of competitiveness calculated with exported goods can predict economic growth. In addition, these indicators are not a mere proxy of the economies’ degree of trade openness since their influence remains when both variables are used together in growth regressions. At the cost of using aggregated data and reducing the provision of information in the indicators of competitiveness, in [48], the authors assembled a database of goods and services in order to calculate different metrics of complexity (fitness). These authors find that services are, on average, more complex than goods; however, the aggregate nature of their data diminishes the forecasting capacity of these indicators in regression analyses.*

**Remark** **4.**
*This is precisely the approach used by the Latin-American group, which establishes the metrics for country and product complexity in terms of two systems of linear difference equations (http://atlas.cid.harvard.edu/learn/glossary). For a critical assessment of this approach see [49].*

**Remark** **5.**
*An indicator of competitiveness is more suitable than another if, on the one hand, it provides a better description of the export data structure and, on the other, if it can explain or predict an economic phenomenon significantly better. This seems to be the case of Endogenous Fitness in comparison with ECI when analyzing world export data. The metrics of Fitness are more consistent with the nested pattern observed in the empirical M_sp_ matrix (highly competitive countries export many products and some of them are very complex, while poorly competitive ones export few products and none of them are complex) [50]. Moreover, the Fitness index seems to be a good predictor of per capita GDP growth in a range of 5–15 years for a large sample of countries (for more details, see Remark 8 and cited references). For a epistolary exchange over these two metrics see [49,51,52,53].*

**Remark** **6.**
*The non-linear nature of the algorithm makes possible that the fitness scores for some regions might converge to zero in real empirical networks [48,54]. With our data, this is the case for only seven states, whose fitness scores are low for n = 50 but practically zero for n = 10,000. However, for the 100th iteration, these scores are very low but still different among them, while for the remaining states, their scores are not meaningfully modified. Furthermore, the states’ rankings do not change for n = 50, 100, 500, or 1000, and when n = 10,000 there are changes only for seven states exhibiting a fitness score extremely close to zero. Consequently, our results are robust to the number of iterations in the following range: 50 ≤ n ≤ 1000. The algorithm is not appropriate before 50 iterations because positive scores have not converged yet, nor is it after 1000 iterations because the rankings of very low fitness states are very volatile.*

**Remark** **7.**
*The Spearman statistic is a rank order correlation; thus, it measures if the observations of two variables tend to have the same relative positions. Because of this, two indicators in a perfect linear association or in any other monotonic relationship (e.g., a logistic curve) have a correlation of 1. In other words, the position between observations, but not their distance, is the only relevant feature captured by this statistic. Therefore, we want to emphasize that, in this paper, we do not assert that with a correlation of 90%, the remaining 10% that corresponds to the discrepancies between the two metrics contains the features needed to explain the superior performance of one indicator over another. Even with a 100% correlation, each metric can exhibit different results when used to infer the evolution of a third variable (e.g., GDP, as in the paper). This is why, even with a very high average of the yearly Spearman correlations, the fitness–income dynamic that we present in several sections of the paper with different metrics is far from identical. In these diagrams, the magnitudes of the fitness scores, and not their rankings, are the predictors of future GDP.*

Another measure of similitude between two vectors is the Cosine Similarity coefficient which measures the cosine of their angle. The resulting value ranges from −1 to 1 with 0 meaning orthogonality, while in-between positive (negative) values signal an intermediate similarity (dissimilarity). The Cosine Similarity statistic is given by the following expression:

$$\;Cosine\;Similarity=\cos\left(\theta \right)=\frac{a\cdot b}{\left|a\right|\left|b\right|}\;$$

where a·b is the dot-product between vector a and vector b, and $\left|a\right|=\sqrt{\sum_{i=1}^{n}a_{i}}$ is the vector norm.

Therefore, the averages of the yearly Spearman correlations and the yearly Cosine Similarity coefficients between the scores of EndoFit and ExoFit during the period 2004–2014 are 0.9843 and 0.8580, respectively; that is, with this alternative statistic, their similitude decreases by 0.1263.

**Remark** **8.**
*Evidence presented in [55,56] shows that the SPS method outperforms traditional tools, used by IMF analysts, for forecasting countries’ growth. Out-of-sample tests are conducted using the mean absolute error (MAE) and the root mean square error (RMSE) as evaluation criteria. With a recently cleaned database coming from raw data of the UN COMTRADE, [56] reports that this non-linear procedure outperforms five-year IMF forecasts by more than 25% in three distinct five-year windows: 2008–2013, 2009–2014, and 2010–2015. It is also important to recognize that the IMF tools are more demanding in terms of data requirements and that such analysis is country-specific. Furthermore, the SPS methodology provides a non-linear criterion of predictive confidence since it asserts that only countries positioned in laminar regimes can exhibit reliable forecasts.*

**Remark** **9.**
*Curiously, the weather forecast with the Method of Analogues has not been a successful endeavor. This is explained by the chaotic nature that this type of time series exhibits in long horizons, especially in micro-areas that are affected by different factors, some of them difficult to measure [57]. *

**Remark** **10.**
*Traditional regression analyses presented in [23,31] with data of Mexican states show that ECI (an exogenous indicator of competitiveness) has a statistical capacity for predicting growth in a span of half a decade. In the first econometric model, this result was obtained defining per capita GDP without oil mining, while in the second model a dummy variable was used to control for states where oil mining represents more than 5% of its GDP (see also Appendix A6).*

**Remark** **11.**
*The non-linear nature of this algorithm creates the possibility that a single product might exert a strong effect on the fitness indicator of every region, Likewise, it is also feasible that some products can be classified as complex for the wrong reasons—that is, because of their rarity and not because they are intrinsically difficult to produce.*

**Remark** **12.**
*In fact, evidence presented in [58] at the country level also shows the existence of development traps. This study highlights that successful countries in the last four decades (e.g., South Korea and Thailand) had to improve their export profile for many years before observing a sustained GDP growth.*

**Remark** **13.**
*If we estimate the complexity of raw petroleum with the ExoFit and the EndoFit metrics, we find that in the former case it occupies the place number 1238 out of a total of 1241 products, while in the latter case it occupies the place number 1156 out of a total of 1159. These places indicate that this industry does not move up in the ranking with a metric using only Mexican data; hence, in both cases, the product is considered of a low complexity. Then, if we calculate EndoFit with tourism, we also find a relatively low place for this industry in the complexity ranking: 1120, that is, not very different from raw petroleum. Paradoxically, the removal of oil from the sample improves the forecasting ability of ExoFit and greatly improves the forecasting ability of EndoFit, yet the inclusion of tourism does not hamper the forecasting ability of EndoFit (ExoFit cannot be calculated since we do not have data for this industry at the world level). Consequently, in this exercise, the node’s centrality is not the factor that modifies the outcome of our performance test when the corresponding industry is excluded from the sample.*

Accordingly, from this preliminary evidence, we can only say that products with a large share in the economy’s export and a low ranking in the complexity index might exert a significant influence in the forecasting procedure used here. Our results indicate that the local bias introduced when using EndoFit can be helpful in capturing the subnational productive structures and for predicting per capita GDP. This is the case when raw petroleum is excluded from the sample, but a similar removal is not necessary in the case of tourism. Perhaps petroleum but not tourism conditions the linkages and evolution of the remaining sectors of the Mexican economy.

**Remark** **14.**
*If the councils managing these funds are composed by a diversified pool of academics, entrepreneurs, analysts, local authorities, and members of civil society, it is more likely that they can reach suitable solutions for the community. The diversity of these councils, their relative independence with respect to lobbying groups, and the use of a decentralized process of decision-making is indispensable for “swarm intelligence” (or crowd-thinking) to work. The main objective of these capital funds should go beyond making profits; thus, they should not be chartered as limited liability corporations. Neither should they be conceived as charity foundations or NGOs with a philanthropic mission; instead, these funds should be financially sustainable despite the fact that they pursue social benefits, as in the Bangladesh’s Grameen Bank and many cooperatives operating around the world. The funding for these councils (or socially oriented venture funds) may come from different sources: government agencies in charge of fostering industry, multilateral banks, or resources derived from interest charged on their credits and from dividends generated by their capital investments.*

**Remark** **15.**
*Moral hazard and adverse selection problems can be ameliorated when the councils of industrial coordination establish stringent filters for potential candidates and when these guarantees have an initial cost and a limited coverage. *

**Remark** **16.**
*Firms can register their projects in these councils with a dual objective. Firstly, they can obtain the right to participate in contests for seed capital and/or credit; the latter can be subsidized temporarily in order to offset the loss of innovation rents due to imitation. Secondly, all projects registered in the council that get funded through private entities can claim an ex-post temporal subsidy when they show to be the source of positive externalities, such as the creation of similar companies and the formation of human capital within the region. These contests are not only schemes providing material incentives but also catalyzers of social change. In contraposition with the inefficiencies generated by subsidies assigned directly by the government bureaucracy, this award system encourages entrepreneurs to innovate and search for solutions to local problems. Besides the material benefits of the award, selected entrepreneurs obtain a social recognition for the externalities they create within the community. This type of recognition not only offsets the material losses due to the generation of spin-offs, but also stimulates the propagation of a social norm where a creative entrepreneur is a synonym of prestige; this is a cultural trait that is not commonly observed in regions exhibiting serious shortcomings.*

**Remark** **17.** *For the Mexican case, the Economic Productivity Unit at the Ministry of the Treasury (Secretaría de Hacienda y Crédito Público, SHCP) commissioned the elaboration of 17 reports on the economic complexity of different Mexican states. These reports present four lists of industries that are not currently competitive in the region but that exhibit particular attributes (whose weights change in each list): (a) the ratio of the product’s export value with respect to Mexico’s total exports, (b) the existing capabilities within the region to produce it, (c) its strategic position in the product space, (d) its complexity. The reader can download these reports in the following URL address: https://www.gob.mx/productividad/documentos/reportes-subnacionales-de-complejidad-economica*.

### Appendix A2. Databases

*Mexican database*: Exports, imports, and formal employment at the state and municipality level. *El Atlas de la Complejidad Económica de México* (http://complejidad.datos.gob.mx/) was elaborated at Harvard’s Center for International Development (CID) with the collaboration of the Laboratorio Nacional de Políticas Públicas (LNPP) at Centro de Investigación y Docencia Económica (CIDE), and the Unidad de Productividad Económica at Secretaría de Hacienda y Crédito Público (SHCP). This database was built with employment and customs information provided by the Instituto Mexicano del Seguro Social (IMSS) and the Sistema de Administración Tributaria (SAT), respectively. Industries (exported products) are defined using the Harmonized System at the four-level digit (HS-4).

*World database*: Exports and imports at the country level. *The Atlas of Economic Complexity* (http://atlas.cid.harvard.edu/engage#data-download) was elaborated at Harvard’s Center for International Development. The raw trade data comes from the United Nations COMTRADE database.

*Tourism information*: “Statistical Compendium of Tourism in Mexico 2016”, whose URL address is the following: http://www.datatur.sectur.gob.mx/SitePages/CompendioEstadistico.aspx.

The estimation of income spent by foreign people in Mexico (TE) at the state level was calculated yearly with the following approximation: $TE_{i,j}=NT_{i,j}*AT_{j}$; *i* =1,…, 32, j=2004,…, 2014, where

$$NT_{i,j}=\mathrm{Number}\;\mathrm{of}\;\mathrm{foreing}\;\mathrm{tourists}\;\mathrm{visiting}\;\mathrm{state}\;i\;\mathrm{in}\;\mathrm{time}\;j\;$$

$$\;\;AT_{j}=\mathrm{National}\;\mathrm{average}\;\mathrm{expenditure}\;\mathrm{of}\;\mathrm{foreign}\;\mathrm{tourism}\;\mathrm{in}\;\mathrm{time}\;j.\;$$

*GDP information*: “Producto interno bruto por entidad federativa”, INEGI, whose URL address is the following: http://www.inegi.org.mx/est/contenidos/Proyectos/SCN/C_Anuales/pib_ef/default.aspx.

*Population information*: “Estimates and projections of the population by federal state”, CONAPO, whose URL address is the following: http://www.conapo.gob.mx/es/CONAPO/Proyecciones_Datos.

### Appendix A3. The Income–Fitness Dynamic Excluding Raw Petroleum

Instead of removing the oil-dependent states of Tabasco and Campeche from the database, we can analyze the required condition for growth predictability by means of the Exogenous Fitness indicator excluding raw petroleum. This *ExoFit-oil* indicator comes from recalculating product complexity from the world database and then obtaining state fitness from the sum of the complexity scores for all products whose revealed comparative advantage is greater than one. As in the other exogenous definitions of fitness, we define the latter coefficient with the regional share of the product in the total of state exports divided by its international share in the total of world exports.

As can be seen from the top panel of Figure A1, the estimated coefficients ${\tilde{D}}_{k}\;$ have relatively low values especially in high income cells. Therefore, we can conclude that the direction of the arrows departing from similar coordinates is very diverse, which discards the forecasting capacity of Exogenous Fitness. It is important to emphasize that in these two plots the vertical axis is defined with per capita GDP excluding oil mining. Moreover, the bottom plot of estimated versors does not show a sole set of coordinates that can be unambiguously described as a *laminar regime*; however, for states with a relatively high fitness, there seems to be certain order. Thus, we can argue that the exogenous variant of the fitness metric does not improve its performance significantly when the exports of raw petroleum are removed from the calculations.

### Appendix A4. The Income–Fitness Dynamic Excluding Oil-Dependent States

The purpose of this section is to conduct some extra sensibility analyses of the income and fitness dynamic. That is, we study the indicators of competitiveness considered in the main text but removing the states of Campeche and Tabasco from the sample. In particular, we want to show whether our predictability assessments remain when keeping raw petroleum in the metric’s calculations and defining income as per capita GDP. As shown in both panels of Figure A2, *ExoFit* does not seem to be a good predictor of income growth, especially for states with middle and high fitness where a *chaotic regime* prevails. In contrast, the dynamic of the *EndoFit* index described in Figure A3 is much more ordered. With the exception of one arrow with very high income and high fitness, the rest of the initial coordinates above a medium level of fitness exhibit a virtuous circle of growth and opportunities. As in the analysis where these two states are included but raw petroleum is excluded, the plot of estimated versors shows a development trap for unproductive states.

Next, in Figure A4, we study the dynamic of the system when using the index of Economic Complexity. In terms of predictability, the results are not very different from those obtained when including the full sample of states and measuring income with per capita GDP excluding oil mining. However, according to the plot of estimated versors, there is a chaotic transition zone between two ordered regimes with contrasting motions. That is, for states with a medium level of complexity, the economy can either move toward a virtuous circle or fall into a development trap. Finally, as suggested by Figure A5, the predictability of growth by means of *EndoFit* with tourism remains practically intact, despite oil mining being included in our measure of aggregate income. All in all, these sensibility exercises indicate that *EndoFit* is a better indicator than *ExoFit* and *ECI*, at least with respect to the condition required for the predictability of the systems dynamic (i.e., the presence of a *laminar regime*).

### Appendix A5. How Sensitive is Endogenous Fitness to a Non-Monetary Normalization Procedure?

The observed changes in the rankings of some states when raw petroleum is excluded or when tourism is included, suggest that the ratio of export value shares could introduce a bias in the measurements of the revealed comparative advantage. Therefore, as a sensibility analysis, we redefine the RCA coefficients by dividing the export value of a good in each state and in Mexico by the corresponding number of inhabitants. This procedure has already been used in the literature of capabilities and development in a network framework [59]. Likewise, it creates a non-monetary indicator of the competitiveness of a product in a given region since it eliminates the effect caused by changes in the price of the good. This can be noticed clearly from the following mathematical expression:

(A1) RCAsp=x(s,p)Pops∑sx(s,p)PopM 

where *x(s*, *p)* is the export value of product “*p*” in state “*s*”; $Pop_{s}$ and $Pop_{M}$ are the population of the state and the whole Mexican population, respectively. As before, we consider the state “*s*” to be competitive in exporting product “*p*”, if $RCA_{sp}\geq 1$.

With this alternative definition of the RCA coefficients, we recalculate the Endogenous Fitness indicators without tourism (*EndoFit~Pop*) and with tourism (*EndoFit + Tourism~Pop*). The average of the yearly Spearman correlations between *EndoFit~Pop* and *EndoFit* is very high (0.9331), and even higher (0.99993) when we compare *EndoFit~Pop* and *EndoFit + Tur~Pop*. We present the rankings evolution for *EndoFit + Tourism~Pop* in Figure A6, where stability is still the norm (with the exception of a non-explained jump in Yucatan for the year 2008). Notice that this procedure moves the oil-dependent states (Campeche and Tabasco) up in the ranking, in comparison to the results of Figure 1, as well as the tourism-dependent states (Quintana Roo and Baja California Sur), in comparison to the results of Figure 8.

In order to judge whether this new specification of RCA provides a better alternative, it has to be a source of reliability for the growth forecasts produced with the SPS methodology. As shown in the top panel of Figure A7, the predictability of the system is good enough in most of the cells of the fitness–income plane, with the exception of a small area in the southeast corner that presents an orange tone. Anew, the versors diagram of the bottom panel shows two areas with clear laminar regimes; however, there is an outlier that corresponds to the trajectory of the state of Campeche. From these results, we can conclude that the *EndoFit* indicator does not improve its performance by adjusting the RCA coefficients with a population variable.

### Appendix A6. Regional Growth Regressions

In this appendix we present the results of a set of growth regressions where we measure productive capabilities with some of the alternative metrics studied in the paper. The main concern here is to analyze whether these indicators exhibit a similar relationship with income growth and, hence, if their corresponding in-sample predictions are close to each other. We would expect to capture a non-linear association in our estimations. The graphical analyses of the SPS procedure (presented in the main text) suggest that for medium and large fitness states the relationship is positive, while for low fitness states such relationship is absent (although, it is negative for the income level).

For testing the presence of a non-linear functional form, we introduce in our regression models the three terms of a cubic polynomial in a step-wise fashion. Then, the statistical significance of the quadratic or cubic terms signals that our predictions of income growth can improve when the non-linear components of the fitness/complexity indicator are taken into account in the econometric model. Likewise, we want to explore whether the alternative indicators have different forecast performances at the aggregate level (e.g., mean square errors, R-squares) and at the state level (point estimates). In other words, we want to find out how similar overall prediction’s errors (or goodness-of-fit) are even if the estimated functional forms for the three variants of models are not alike, and whether or not growth forecasts for particular states vary substantially when we use the different metrics (*EndoFit*, *ExoFit* and *Eci*).

We run econometric models for the annualized growth in the period 2004–2014 with a cross-section of 32 states, and for the annualized growth observed in two periods (2010–2014 and 2004–2008) with a panel of 64 observations. We estimate the former models with OLS regressions and the latter ones with random and fixed effects regressions. As commonly done in these statistical exercises, we use control variables defined at the beginning of each period: per capita GDP (in logarithms, as a measure of initial economic activity), population (in logarithms, as a measure of state size) and education (average schooling for individuals with at least 15 years of age). We also produce estimations correcting for oil-dependency. This is done through three different mechanisms: eliminating raw-petroleum from the database and recalculating the metrics (*EndoFit~Oil*, *ExoFit~Oil*), introducing dummy variables for oil-dependent states (Tabasco and Campeche), and removing those states from the database.

#### Appendix A6.1. Estimation Results for Endogenous Fitness

Table A1 shows the regional growth regressions with Endogenous Fitness. In Models 1–4 we analyze growth for the period 2004–2014 by means of OLS regressions. In Models 5–8 we analyze growth for two periods of half a decade, 2010–2014 and 2004–2008, by means of panel models with random effects. The main results derived from Table A1 are the following. (i) The logarithm of per capita GDP at the beginning of the period has a negative and statistically significant coefficient in most of these models, which implies that the hypothesis of relative convergence holds. (ii) For Models 1–4, we did not find a non-linear statistical relationship between Endogenous Fitness (*EndoFit*) and growth; the only significant linear term has a negative coefficient; the largest adjusted-R^2^ is obtained when we introduce a dummy variable for identifying the state of Tabasco. (iii) For Models 5–8, there is a non-linear relationship since the three terms of the cubic polynomial are statistically significant in the last two models; again the best fit is obtained when the dummy for Tabasco is included as a control variable, although, with a much lower R^2^ (overall and within); in fact, the last model is the only one with overall significance according to the Wald Chi statistic. (iv) The two control variables, population and education, are not statistically significant in any of these four models. (v) The functional form for Endogenous Fitness has a concave-then-convex shape since the quadratic term is negative and the cubic term is positive; in certain range of the capabilities indicator, this form can be consistent with the pattern observed in the data according with our SPS analysis.

**Table A1:** Regional growth regressions with Endogenous Fitness, 2004–2014. Dependent variable: annualized growth rates for periods of 11 and 5 years (per capita gross domestic product, inflation adjusted).

	Model 1	Model 2	Model 3	Model 4	Model 5	Model 6	Model 7	Model 8
Log(GDP_p_)	−0.0173255 *** (0.0039995)	−0.0175403 *** (0.003894)	−0.0183869 *** (0.0039537)	−0.0200634 *** (0.0037319)	−0.0144554 (0.0104048)	−0.015769 (0.0101421)	−0.017082 * (0.0097757)	−0.0186439 ** (0.0091743)
EndoFit	−0.0023644 (0.0014368)	−0.0073397 ** (0.003434)	0.0025256 (0.0095901)	0.0060215 (0.009006)	0.0007554 (0.0012829)	0.0047724 * (0.0028566)	0.0197269 ** (0.0089051)	0.0213824 ** (0.009532)
EndoFitSq		0.0005771 (0.0003638)	−0.0022698 (0.0026109)	−0.0031274 (0.0024451)		−0.0004149 (0.0002832)	−0.0046572 ** (0.0021766)	−0.0049994 ** (0.0023128)
EndoFitCu			0.0002034 (0.0001847)	0.0002612 (0.0001728)			0.0002908 ** (0.0001357)	0.0003113 ** (0.0001439)
Log(Pop)	0.0066581 * (0.003531)	0.0079357 ** (0.003529)	0.0056816 (0.0040674)	0.0044504 (0.0038024)	0.0004058 (0.003067)	−0.0014418 (0.0035538)	−0.0039679 (0.0038233)	−0.0046521 (0.0041124)
Edu	0.0104072 *** (0.0032049)	0.0114063 *** (0.0031815)	0.009516 ** (0.0036038)	0.0090339 ** (0.0033415)	0.0015736 (0.0024411)	0.0008942 (0.0023368)	−0.0000913 (0.0022483)	−0.0000355 (0.0021719)
DTabasco				0.023672 ** (0.010386)				0.0203133 *** (0.0066683)
Constant	0.0384033 (0.0840078)	0.0162002 (0.0829336)	0.0709979 (0.0964312)	0.1101432 (0.0908727)	0.1645297 (0.1274021)	0.210625 (0.1337973)	0.2679194 ** (0.1360862)	0.2942927 ** (0.137251)
Obs.	32	32	32	32	64	64	64	64
R2	0.4606	0.4893	0.4934	0.5662	0.1953 0.0637	0.1918 0.2251	0.2252 0.2578	0.2577 0.2649
Statistic	7.62	6.94	6.03	6.78	2.59	4.63	9.27	16.35
Prob. > Stat.	0.0003	0.0003	0.0005	0.0002	0.6279	0.4626	0.1587	0.0221

**Notes:** The postfixes *Sq* and *Cu* indicate that the corresponding variable has been raised to the 2nd and 3rd power, respectively; coefficients’ standard deviations are defined in the parenthesis; * *p* < 0.1, ** *p* < 0.05, *** *p* < 0.01; DTabasco is a dummy variable identifying the state of Tabasco; R2 is adjusted for Models 1–4, and their significance is tested with an F-statistic; errors’ standard deviations are not corrected for heteroscedasticity due to limited degrees of freedom in some of these models; R2 is the overall value (top line) and the within value (bottom line) for Models 5–8, and their significance is tested with the Wald Chi statistic; errors’ standard deviations are robust to heteroscedasticity.

#### Appendix A6.2. Estimation Results for Exogenous Fitness and Economic Complexity

Despite that the R-square is substantially larger in the OLS regressions and that these models pass the test of overall significance, we can argue that the statistical results have a low power due to the limited number of observations. Furthermore, in contrast to previous findings of this literature, the sign of the linear term of the complexity/fitness variable is negative (Model 2). Consequently, in the rest of the analysis, we only present panel data models with random and fixed effects for two periods of five years, 2010–2014 and 2004–2008. In Table A2 we exhibit random effects estimates for alternative indicators of productive capabilities: Exogenous Fitness (*ExoFit*) in Models 1–4 and Economic Complexity (*Eci*) in Models 5–8.

The most salient results of Table A2 are the following. (i) The convergence hypothesis holds for models that use the whole set of independent variables. (ii) For both indicators, the linear functional form is not the proper statistical association between growth and productive capabilities. (iii) When a dummy variable for Tabasco is included in the model, its estimated coefficient is always statistically significant. (iv) The cubic term of *ExoFit* is not statistically significant and the functional shape of this variable is concave, while the quadratic term of *Eci* is not significant and the cubic term presents the opposite sign to that found for *EndoFit*. (v) With regard to the overall fitting, the R-square is largest for *ExoFit* (0.4039), followed by *Eci* (0.3258) and then by *EndoFit* (0.2577). (vi) In relation to the fitting within the units of observation, the R-square is largest for *Eci* (0.3260), followed by *EndoFit* (0.2649) and then by *ExoFit* (0.1947). In brief, from the first two tables we can conclude that there seems to be a non-linear relationship between the indicator of productive capabilities and growth. However, the fitted functional forms are different and this might imply that their predictive capabilities could not be the same. It is important to mention that if the purpose of the estimated equation is to forecast future growth for a specific set of values of the independent variables, the model including *Eci* is the best candidate according to the within value of R-square; however, if the purpose is to estimate growth elasticities when only productive capabilities vary, the model including *ExoFit* is the best option as suggested by the overall value of R-square.

#### Appendix A6.3. Estimated Results for Oil-dependence Corrected Regressions

In this section we proceed to show the results of oil-corrected regressions. This is due to the fact that the dummy for Tabasco produces a significant coefficient in the two previous tables and a better fitting. Moreover, from the main text, we know that the criterion for predictability is better met in our SPS exercises when we make this sort of modifications in our calculations. In Models 1–4 of Table A3 we apply these interventions to the regressions with Endogenous Fitness, in Models 5–6 to the regressions with Exogenous Fitness, and in Models 7–8 to the regressions with Economic Complexity. This can be done with different procedures: introducing a dummy variable for both oil-dependent states (Campeche and Tabasco) like in Models 1, 5 and 7; using a dummy variable only for Tabasco like in Models 4 and 6; redefining the indicators by removing raw petroleum, like in Models 2, 4 and 6; or excluding from the sample the states of Campeche and Tabasco like in Models 3 and 8.

The highlights of these regressions results are the following. (i) The polynomial of degree three is still statistically significant for Endogenous Fitness with a concave-then-convex shape, unless the oil-dependent states are removed from the sample (Model 3); (ii) Exogenous Fitness now exhibits a linear relationship with growth, while the same non-linear relationship for Economic complexity prevails, even if Tabasco and Campeche are removed from the sample (Model 8). (iii) The dummy for oil-dependent states does not have a statistically significant coefficient. (iv) The regressions with the reduced sample yield a much lower goodness-of-fit (within value of the R-square), and a statistically significant but negative sign for the schooling variable; contrary to our priors. (v) The best fitting (within value) of the oil-corrected regressions diminishes for *EndoFit*, increases marginally for *Eci* and improves slightly for *ExoFit*, although in the latter case with a linear relationship. (vi) Similar regressions, not presented here, were run with per capita GDP without oil-mining but the conclusions did not change qualitatively in terms of the signs and statistical significance of the coefficients, although they exhibited a much lower fitting.

All in all, the random effects regressions in Table A1, Table A2 and Table A3 show that the three indicators of capabilities do not fit the subnational data with the same functional relationships, despite of the fact that there is a high Spearman correlation between the different metrics. This finding seems to coincide with the different degrees of predictability observed in the SPS procedure. Nonetheless, some of these estimations are capable of describing the non-linear relationship with growth observed in the Mexican data even when controls are introduced in the regression analysis. However, this does not happen for the estimations with *ExoFit* when the model is corrected for oil-dependence. In this case, a linear functional form prevails and its estimated coefficient has a positive sign, which implies that any increase in productive capabilities of certain size yields a uniform improvement in 5-years income growth.

**Table A2:** Regional growth regressions with Exogenous Fitness and Economic Complexity, 2004–2014. Dependent variable: annualized growth rates for two periods of five years (per capita gross domestic product, inflation adjusted).

	Model 1	Model 2	Model 3	Model 4	Model 5	Model 6	Model 7	Model 8
Log(GDP_p_)	−0.0165319 * (0.0089899)	−0.0148992 * (0.0086574)	−0.0140956 (0.0089671)	−0.0149223 ** (0.0069951)	−0.0143415 (0.0088832)	−0.0139801 (0.0095308)	−0.0142191 (0.0096114)	−0.015892 * (0.0087936)
ExoFit	0.0102867 ** (0.0041612)	0.0209965 ** (0.0092185)	0.0288682 (0.02022)	0.0489177 *** (0.0171303)				
ExoFitSq		−0.0036485 (0.0024707)	−0.0100866 (0.0140177)	−0.0214376 * (0.0124)				
ExoFitCu			0.0013129 (0.0026414)	0.0032552 (0.0023666)				
Eci					0.0089863 ** (0.0038962)	0.0088548 ** (0.0043927)	0.0159825 ** (0.0073132)	0.0186952 ** (0.007804)
EciSq						0.0000689 (0.0042466)	0.0030141 (0.0035541)	0.0036416 (0.0037432)
EciCu							−0.0064342 * (0.0034704)	−0.0075883 ** (0.0036262)
Log(Pop)	−0.0055626 (0.0043632)	−0.0050557 (0.0043232)	−0.0049136 (0.0043282)	−0.0066017 (0.004778)	−0.0022973 (0.0042544)	−0.0021 (0.0042271)	−0.0020939 (0.0045071)	−0.0027713 (0.0048939)
Edu	0.0001228 (0.002164)	−0.0001628 (0.0021382)	−0.0001617 (0.0021671)	−0.0003775 (0.0020473)	0.0000613 (0.0023212)	0.0001785 (0.0022342)	0.0000171 (0.0020751)	8.63 × 10 ^−6^ (0.0019351)
DTabasco				0.0358943 *** (0.007187)				0.0264273 *** (0.0079933)
Constant	0.2784872 ** (0.1321611)	0.2498717 * (0.1276447)	0.2366058 * (0.1325907)	0.2633407 ** (0.1268572)	0.212915* (0.1257222)	0.2049188 (0.1310565)	0.208004 (0.1401495)	0.2357233 (0.144123)
Obs.	64	64	64	64	64	64	64	64
R2	0.2497 0.2733	0.2895 0.2889	0.2970 0.2560	0.4039 0.1947	0.2806 0.2257	0.2830 0.2216	0.2712 0.3279	0.3258 0.3260
Wald Chi	8.33	11.87	12.71	44.80	14.14	14.02	11.11	23.70
Prob > Chi^2^	0.0802	0.0367	0.0479	0.0000	0.0069	0.0155	0.0849	0.0013

**Notes:** The postfixes *Sq* and *Cu* indicate that the corresponding variable has been raised to the 2nd and 3rd power, respectively; coefficients’ standard deviations are defined in the parenthesis; * *p* < 0.1, ** *p* < 0.05, *** *p* < 0.01; random effects estimations with two periods of five years: 2010–2014 and 2004–2008; R2 is the overall value (top line) and the within value (bottom line); the significance of the model is measured with the Wald Chi statistic; errors’ standard deviations are robust to heteroscedasticity; DTabasco is a dummy variable identifying the state of Tabasco.

**Table A3:** Growth regressions corrected for oil-dependence, 2004–2014. Dependent variable: annualized growth rates for two periods of five years (per capita gross domestic product, inflation adjusted).

	Model 1	Model 2	Model 3	Model 4	Model 5	Model 6	Model 7	Model 8
Log(GDP_p_)	−0.0145268 (0.0136423)	−0.0164121 * (0.0099913)	0.0210509 *** (0.0073088)	−0.0178092 * (0.0095139)	−0.0170732 (0.0142267)	−0.0170839 ** (0.0074607)	−0.0128544 (0.0129291)	0.0182204 *** (0.0066289)
EndoFit	0.0186449 * (0.009982)		0.0061016 (0.006722)	.				
EndoFitSq	−0.0044425 * (0.0023349)		−0.0022026 (0.0018052)					
EndoFitCu	0.0002783 * (0.0001437)		0.0001587 (0.0001248)					
EndoFit~Oil		0.0161024 ** (0.0073745)		0.0173203 ** (0.0078194)				
EndoFit~OilSq		−0.0036825 ** (0.001652)		−0.0039153 ** (0.0017375)				
EndoFit~OilCu		0.0002192 ** (0.0000964)		0.0002321 ** (0.0001011)				
ExoFit					0.0345633 * (0.0209359)			
ExoFitSq					−0.0125525 (0.0128991)			
ExoFitCu					0.001683 (0.0023846)			
ExoFit~Oil						0.0458676 ** (0.0200682)		
ExoFit~OilSq						−0.0177393 (0.0137968)		
ExoFit~OilCu						0.0024239 (0.0026225)		
Eci							0.0155941 * (0.0080645)	0.0106833 ** (0.0049313)
EciSq							0.0066555 (0.0039978)	0.0066555 (0.0054479)
EciCu							−0.006685 * (0.0035449)	−0.0089164 * (0.0048503)
Log(Pop)	−0.0036141 (0.0041484)	−0.0026159 (0.0035318)	0.0019717 (0.0030873)	−0.003159 (0.003765)	−0.0058348 (0.0057272)	−0.0072285 (0.0052079)	−0.0019833 (0.0047752)	−0.0000662 (0.002424)
Edu	−0.0003489 (0.0022064)	0.0004749 (0.0022924)	−0.0036114 * (0.0018429)	0.0005514 (0.0022155)	−0.0000164 (0.0021688)	−0.0003506 (0.0019459)	−0.0002263 (0.002007)	−0.0043241 ** (0.001863)
DOil	−0.0075064 (0.0256846)				0.0093994 (0.0299999)		−0.0048651 (0.0307612)	
DTabasco				0.0182323 *** (0.0067492)		0.0239798 *** (0.0059947)		
Constant	0.2363029 (0.1890923)	0.2374505 * (0.1331446)	−0.2198894 ** (0.1031526)	0.259925 * (0.1341542)	0.2796539 (0.214558)	0.2969323 ** (0.1376459)	0.1928784 (0.1856774)	−0.1548453 * (0.08371)
Obs.	64	64	60	64	64	64	64	60
R2	0.2407 0.2475	0.2156 0.1976	0.2768 0.0232	0.2433 0.2054	0.2916 0.2585	0.3602 0.2642	0.2721 0.3361	0.2927 0.0843
Wald Chi	10.92	10.19	12.82	19.78	17.97	29.63	11.03	15.93
Prob > Chi^2^	0.1423	0.1168	0.0459	0.0061	0.0121	0.0001	0.1371	0.0141

**Notes:** The postfixes *Sq* and *Cu* indicate that the corresponding variable has been raised to the 2nd and 3rd power, respectively; coefficients’ standard deviations are defined in the parenthesis; * *p* < 0.1, ** *p* < 0.05, *** *p* < 0.01; random effects estimation with two periods of five years: 2010–2014 and 2004–2008; R2 is the overall value (top line) and the within value (bottom line); the significance of these models is measured with the Wald Chi statistic; errors’ standard deviations are robust to heteroscedasticity in all models but in 3 and 8 due to limited degrees of freedom; the states of Campeche and Tabasco are removed from the sample in Models 3 and 8; DTabasco is a dummy variable identifying the state of Tabasco; DOil is a dummy variable identifying the states of Campeche and Tabasco.

#### Appendix A6.4. Estimation Results for Models with Fixed Effects

The significance of the dummy variable for the state of Tabasco and the drastic change in the within values of the R-square when the oil-dependent states are removed from the sample, suggest that a fixed effects model can be suitable for these variables and the Mexican database. In fact when we apply the Hausman test, we reject the null hypothesis that the state-fixed effects are not needed for our three indicators of productive capabilities. Besides this result, from Table A4 we infer the following observations. (i) The functional form for productive capabilities varies depending on the indicator used; for *EndoFit* the same concave-then-convex relationship prevails; however, for *ExoFit* none of the terms in the cubic polynomial is statistically significant, while for *Eci* there is a positive linear relationship. (ii) The education variable always presents a positive and statistically significant coefficient, while the population variable (in logarithms) is also significant but its coefficient has a negative sign. (iii) The overall R-squares in these models are much smaller than in the random effects estimates, but the within values are much larger. (iv) The model with the best predictive capabilities (within values) is the one including *EndoFit* (0.6022), followed by *Eci* (0.5314) and then by *ExoFit* (0.4850); however, in the last model the associated linear term is positive but not statistically significant.

Consequently, the empirical evidence is very strong in favor of different functional forms for each indicator of productive capabilities in growth regressions. Even more, if these models are used for prediction purposes, then the fixed effects regression with Endogenous Fitness seems to offer the best alternative (according to the within values of the R-square). This result coincides with the findings of the non-linear procedure implemented in the main text of this paper, despite of the fact that we used a non-parametric methodology.

**Table A4:** Regional growth regressions with fixed effects, 2004–2014. Dependent variable: annualized growth rates for periods of 11 and 5 years (per capita gross domestic product, inflation adjusted).

	Model 1	Model 2	Model 3	Model 4	Model 5
Log(GDP_p_)	−0.0856704 *** (0.0181986)	−0.0798948 *** (0.0201228)	−0.0786688 *** (0.0195105)	−0.0876209 ** (0.0319398)	−0.0998928 *** (0.0204312)
EndoFit	0.0461811 *** (0.0152243)				
EndoFitSq	−0.0077373 ** (0.0029663)				
EndoFitCu	0.000408 ** (0.0001807)				
ExoFit		−0.0111011 (0.0567444)	0.0142936 (0.011174)		
ExoFitSq		0.023465 (0.0367806)			
ExoFitCu		−0.004571 (0.0063557)			
Eci				0.0191758 * (0.0106878)	0.0164638 ** (0.0077017)
EciSq				0.0045546 (0.0088639)	
EciCu				−0.0024511 (0.0063875)	
Log(Pop)	−0.1520162 *** (0.0455606)	−0.1467475 *** (0.0503463)	−0.1524682 *** (0.0483571)	−0.1218414 ** (0.0499547)	−0.1253733 ** (0.0469399)
Edu	0.0103553 *** (0.0037082)	0.0110999 ** (0.0041726)	0.0116899 *** (0.0039414)	0.0094653 ** (0.0046066)	0.0106828 *** (0.0037743)
Constant	3.142839 *** (0.7072579)	3.000767 *** (0.7813192)	3.06343 *** (0.7554937)	2.737751 *** (0.8476219)	2.923602 *** (0.7213618)
Obs.	64	64	64	64	64
R2	0.0009 0.6022	0.0021 0.4985	0.0029 0.4850	0.0011 0.5362	0.0029 0.5314
F Statistic	6.56	4.31	6.59	5.01	7.94
Prob > Stat	0.0003	0.0038	0.0007	0.0016	0.0002
Hausman	21.36	17.18	14.54	n.a.	22.39
Prob > Stat	0.0016	0.0086	0.0058	n.a.	0.0002

**Notes:** The postfixes *Sq* and *Cu* indicate that the corresponding variable has been raised to the 2nd and 3rd power, respectively; coefficients’ standard deviations are defined in the parenthesis; * *p* < 0.1, ** *p* < 0.05, *** *p* < 0.01; R2 is the overall value (top line) and the within value (bottom line); n.a. = model fitted on these data fails to meet the asymptotic assumptions of the Hausman test; the significance of these models is measured with the F statistic.

#### Appendix A6.5. Predicted Growth with Different Metrics

In this section we analyze adjusted growth and elasticities for the three metrics when the models’ coefficients are estimated with random effects. We do not use the estimates of the fixed effects models since we rejected the significance of *ExoFit*, and we want to illustrate how elasticities can vary depending on the inferred functional form for productive capabilities. Table A5 presents the models used for in-sample prediction in each category of indicator. These are selected from Table A1 and Table A2 considering the largest number of statistically significant coefficients in nested models. According to the Breusch & Pagan Langrange multiplier test, random effects are preferred over OLS (see Row 13 in Table A5). Although the three metrics yield a similar mean predicted growth for the period 2010–2014 (i.e., for just one of the two periods considered in the estimation procedure), forecasts are slightly less erratic for *ExoFit* and *Eci* since the averages of their standard deviations are slightly lower than that observed for *EndoFit* (see Rows 14 and 15).

In relation with the models’ predictive accuracy, we can say that *Eci* has the largest R-square (within values) but *ExoFit* provides the smallest mean square errors for the in-sample forecasts for the two periods (see Rows 11 and 16). Because of the t-tests presented in Table A6, we cannot reject the hypothesis that the mean growth forecasts are identical when only the period 2010–2014 is considered, irrespectively of the pair of indicators analyzed (caveat: these averages are very similar by construction when the two periods are included in the calculation). However, when we compare mean square errors, the accuracy of the fitting does not seem to be identical between *EndoFit* and *ExoFit*. Moreover, we find that the forecasts are equally erratic only when we make a comparison between *EndoFit* and *Eci*, but not in the other two cases. Finally, we fail to reject mean equality for our calculations of elasticities in *EndoFit versus ExoFit*, and in *ExoFit versus Eci*. In general, these overall forecasting results indicate that there are statistical difference between *ExoFit* and the other two metrics.

**Table A5:** Average fitting of predicted growth, 2004–2014. Dependent variable: annualized growth rates for two periods of five years (per capita gross domestic product, inflation adjusted).

		Model A1.8 (*EndoFit*)	Model A2.4 (*ExoFit*)	Model A2.8 (*Eci*)
(1)	Log(GDP_p_)	−0.0161203 ** (0.0078185)	−0.0136732 ** (0.0059063)	−0.0138542 ** (0.0064373)
(2)	EndoFit	0.0178243 ** (0.0079498)		
(3)	EndoFitSq	−0.0043577 ** (0.0020221)		
(4)	EndoFitCu	0.0002745 ** (0.0001277)		
(5)	ExoFit		0.024116 *** (0.008316)	
(6)	ExoFitSq		−0.0053179 * (0.002752)	
(7)	Eci			0.0170175 *** (0.0065092)
(8)	EciCu			−0.0058726 * (0.0033072)
(9)	DTabasco	0.0183141 *** (0.0062984)	0.0272618 *** (0.0069967)	0.0244084 *** (0.0066153)
(10)	Constant	0.197893 ** (0.0881094)	0.1586653 ** (0.0655722)	0.1737042 ** (0.0732403)
(11)	R2	0.2615 0.2089	0.3570 0.1772	0.3414 0.2233
(12)	Wald Chi	16.28 ***	16.80 ***	18.13 ***
(13)	Prob > chibar2 (Breush & Pagan)	0.0028	0.0125	0.0046
(14)	Mean adjusted growth, 2010–2014	0.0180172 (0.0092687)	0.0180025 (0.0100728)	0.0176993 (0.0104244)
(15)	Mean standard deviation of adjusted growth, 2010–2014	0.0046942 (0.0031006)	0.0039256 (0.002623)	0.0043876 (0.0023375)
(16)	Mean square error, 2004–2008 & 2010–2014	0.0002025 (0.0002549)	0.0001755 (0.000223)	0.0001803 (0.0002412)

**Notes:** The postfixes *Sq* and *Cu* indicate that the corresponding variable has been raised to the 2nd and 3rd power, respectively; standard deviations for coefficients and variables are defined in the parenthesis of Rows 1–10 and 14–16, respectively; * *p* < 0.1, ** *p* < 0.05, *** *p* < 0.01; random effects estimations for two periods of five years: 2010–2014 and 2004–2008; R2 is the overall value (top line) and the within value (bottom line); the significance of these models is measured with the Wald Chi statistic; *p*-value < 0.05 in Row 13 indicates that null of no random effects (OLS) is rejected; mean values in Rows 14 and 15 are defined only for the period 2010–2014; in Row 16 square errors are defined with the difference between observed and adjusted growth in the two periods; errors’ standard deviations are robust to heteroscedasticity.

**Table A6:** *t*-tests for mean differences of growth predictions, 2010–2014.

		*EndoFit* versus *ExoFit*	*EndoFit* versus *Eci*	*ExoFit* versus *Eci*
(1)	Growth forecast: *p*-value	0.9841	0.7828	0.6976
(2)	St.Dev. forecast: *p*-value	0.0001	0.2740	0.0772
(3)	Square errors: *p*-value	0.0443	0.1297	0.6787
(4)	Elasticities: *p*-value	0.0634	0.9618	0.0804

**Notes:** These tests are applied to the adjusted values obtained from regressions of Table A5; only the annualized growth for the period 2010–2014 is considered here; alternative hypothesis: means are different; predicted values come from using xb; errors are defined with the compose residuals: ui + eit.

Next, Figure A8 and Figure A9 show that there are important differences in predicted values for specific Mexican states. These are more notorious between *EndoFit* and *Eci* (top panel in Figure A8) than between *ExoFit* and *Eci* (bottom panel) since in the former comparison many of the point estimates are farther away from the 45° line (e.g., 28, 24, 20, 18, 22, 17, 7). Then, if we calculate arch elasticities for predicted growth with an increase of 10% in the states’ fitness/complexity, it becomes evident that *ExoFit* produces relatively similar elasticities and most of them positive (see the top panel of Figure A9). In contrast, the presence of the cubic term in the polynomials of *EndoFit* and *Eci* generates two large spikes of opposite sign in each case, although in different states. However, *Eci* produces more elasticity variation across states and 8 negative values, while *EndoFit* has only 3 (see the bottom panel of Figure A9).

Our objective in this appendix is not to provide an assessment on which metric produces more accurate predictions using traditional regressions analyses. We are only interested in showing that these forecasts differ depending on which complexity/fitness indicator is considered as independent variable. In the random effects models, one can observe different goodness-of-fit at the aggregate level, yet the differences at the state level are more striking. Not only point estimates are very different for several states, but there are also important discrepancies in the calculated arch elasticities of particular cases. These results are mainly a consequence of inferring different functional forms in the relationship between complexity/fitness and income growth. Presumably, these contrasting results will be more notorious with the state-fixed effects models since only *EndoFit* keeps a non-linear relationship. In models with *ECI* such relationship becomes linear, while *ExoFit* is not statistically significant in the corresponding regression analysis.

## References

[1] Imbs J., Wacziarg R. (2003). Stages of diversification. Am. Econ. Rev..

[2] Klinger B., Lederman D. (2006). Diversification, Innovation, and Imitation inside the Global Technological Frontier.

[3] Bustos S., Gómez C., Hausmann R., Hidalgo C.A. (2012). The dynamics of nestedness predicts the evolution of industrial ecosystems. PLoS ONE.

[4] Hidalgo C.A., Hausmann R. (2009). Building blocks of economic complexity. Proc. Natl. Acad. Sci. USA.

[5] List F. (1909). The National System of Political Economy.

[6] Gerschenkron A. (1962). Economic Backwardness in Historical Perspective: A Book of Essays.

[7] Akamatsu K. (1962). A Historical Pattern of Economic Growth in Developing Countries. Dev. Econ..

[8] Kuznets S. (1966). Modern Economic Growth.

[9] Lall S. (2000). The technological structure and performance of developing country manufactured exports. Oxf. Dev. Stud..

[10] Lin J.Y., Stiglitz J.E., Lin J.Y., Patel E. (2013). From flying geese to leading dragons: New opportunities and strategies for structural transformation in developing countries. The Industrial Policy Revolution II. Africa in the 21st Century.

[11] Rosenstein-Rodan P.N. (1943). Problems of industrialization of Eastern and South-eastern Europe. Econ. J..

[12] Prebisch R. (1950). The Economic Development of Latin America and Its Principal Problems.

[13] Hirschman A.O. (1958). The Strategy of Economic Development.

[14] Nelson R., Winter S.G. (1982). An Evolutionary Theory of Economic Change.

[15] Alcouffe A., Kuhn T.J. (2004). Schumpeterian endogenous growth theory and evolutionary economics. J. Evol. Econ..

[16] Pasinetti L. (1981). Structural Change and Economic Growth: A Theoretical Essay on the Dynamics of the Wealth of Nations.

[17] Thirwall A.P. (2002). The Nature of Economic Growth. An Alternative Framework for Understanding the Performance of Nations.

[18] Hausmann R., Hidalgo C.A., Bustos S., Coscia M., Simoes A., Yildirim M. (2013). The Atlas of Economic Complexity. Mapping Paths to Prosperity.

[19] Hausmann R., Hidalgo C.A. (2011). The network structure of economic output. J. Econ. Growth.

[20] Tacchella A., Cristelli M., Caldarelli G., Gabrielli A., Pietronero L. (2012). A new metric for countries’ fitness and products’ complexity. Nat. Sci. Rep..

[21] Hidalgo C.A., Klinger B., Barabási A.L., Hausmann R. (2007). The product space conditions the development of nations. Science.

[22] Caldarelli G., Cristelli M., Gabrielli A., Pietronero L., Scala A., Tacchella A. (2012). A Network Analysis of Countries’ Export Flows: Firm Grounds for the Building Blocks of the Economy. PLoS ONE.

[23] Castañeda G. (2018). Complejidad económica, estructuras productivas regionales y política industrial. Rev. Econ. Mex..

[24] Hausmann R., Cheston T., Santos M.A. (2015). La Complejidad Económica de Chiapas: Análisis de Capacidades y Posibilidades de Diversificación Productive.

[25] Freitas E.E., Andrade E. (2015). Diversificação e sofisticação das exportações: Uma aplicação do product space aos dados do Brasil. Rev. Econ. Nordeste.

[26] O’Clery N., Lora E. (2016). City Size, Distance and Formal Employment Creation.

[27] Borda G.L. (2017). Índice de Complejidad Económica para los Departamentos de Colombia, Evolución 2012–2015.

[28] Operti F.G., Pugliese E., Andrade J.S., Pietronero L., Gabrielli A. (2018). Dynamics in the fitness-income plane: Brazilian states vs world countries. PLoS ONE.

[29] Reynolds C., Agrawal M., Lee I., Zhan C., Li J., Taylor P., Mares T., Morrison J., Angelakis N., Roos G. (2018). A sub-national economic complexity analysis of Australia’s states and territories. Reg. Stud..

[30] Gao J., Zhou T. (2018). Quantifying China’s regional economic complexity. Phys. A Stat. Mech. Appl..

[31] Chávez J.C., Mosqueda M.T., Gómez-Zaldívar M. (2017). Economic complexity and regional growth performance: Evidence from the Mexican economy. Rev. Reg. Stud..

[32] Thurner S., Klimek P., Hanel R. (2010). Schumpeterian economic dynamics as a quantifiable minimum model of evolution. New J. Phys..

[33] Schumpeter J.A. (1934). The Theory of Economic Development: An Inquiry into Profits, Capital, Credit, Interest, and the Business Cycle.

[34] Klimek P., Hausmann R., Thurner S. (2012). Empirical confirmation of creative destruction from world trade data. PLoS ONE.

[35] Cristelli M., Gabrielli A., Tacchella A., Caldarelli G., Pietronero L. (2013). Measuring the intangibles: A metric for the economic complexity of countries and products. PLoS ONE.

[36] Cristelli M., Tacchella A., Pietronero L. (2015). The heterogeneous dynamics of economic complexity. PLoS ONE.

[37] Lorenz E.N. (1969). Atmospheric predictability as revealed by naturally occurring analogues. J. Atmos. Sci..

[38] Morrison G., Buldryev S.V., Imbruno M., Doria-Arrieta O.A., Rungi A., Riccaboni M., Pammolli F. (2017). On economic complexity and the fitness of nations. Nat. Sci. Rep..

[39] Farole T., Akinci G. (2011). Special Economic Zones: Progress, Emerging Challenges, and Future Directions.

[40] Hausmann R., Obach J., Santos M.A. (2017). Special Economic Zones in Panama: Technology Spillovers from a Labor Market Perspective.

[41] Castañeda G., Chávez-Juárez F., Guerrero O.A. (2018). How do governments determine policy priorities? Studying development strategies through spillover networks. J. Econ. Behav. Organ..

[42] Castañeda G., Chávez-Juárez F. (2017). The Incidence of Culture, Governance and Economics on the Countries’ Development through an Analysis of Coupled Networks.

[43] Romer P. (1990). Endogenous technological change. J. Polit. Econ..

[44] Aghion P., Howitt P. (1992). A model of growth through creative destruction. Econometrica.

[45] Aghion P., Howitt P. (1998). Endogenous Growth Theory.

[46] Grossman G.M., Helpman E. (1991). Innovation and Growth in the Global Economy.

[47] Zaccaria A., Cristelli M., Tacchella A., Pietronero L. (2014). How the taxonomy of products drives the economic development of countries. PLoS ONE.

[48] Stojkoski V., Utkovski Z., Kocarev L. (2016). The impact of services on economic complexity: Service sophistication as route for economic growth. PLoS ONE.

[49] Pietronero L., Cristelli M., Gabrielli A., Mazzilli D., Pugliese E., Tacchella A., Zaccaria A. (2017). Economic complexity: ’Buttarla in caciara’ vs a constructive approach. arXiv.

[50] Mariani M.S., Vidmer A., Medo M., Zhang Y.-C. (2015). Measuring economic complexity of countries and products: Which metric to use?. Eur. Phys. J. B.

[51] Albeaik S., Kaltenberg M., Alsaleh M., Hidalgo C.A. (2017). Improving the economic complexity index. arXiv.

[52] Albeaik S., Kaltenberg M., Alsaleh M., Hidalgo C.A. (2017). 729 new measures of economic complexity (Addendum to improving the economic complexity index). arXiv.

[53] Gabrielli A., Cristelli M., Mazzilli D., Tacchella A., Zaccaria A., Pietronero L. (2017). Why we like the ECI+ algorithm. arXiv.

[54] Pugliese E.A., Zaccaria A., Pietronero L. (2016). On the convergence of the fitness-complexity algorithm. Eur. Phys. J. Spec. Top..

[55] Cristelli M., Tacchella A., Cader M., Roster K., Pietronero L. (2017). On the Predictability of Growth.

[56] Tacchella A., Mazzilli D., Pietronero L. (2018). A dynamical systems approach to gross domestic product forecasting. Nat. Phys..

[57] Sylos-Labini F. (2016). Science and the Economic Crisis. Impact on Science, Lessons from Science.

[58] Pugliese E., Chiarotti G.L., Zaccaria A., Pietronero L. (2017). Complex economies have a lateral escape from the poverty trap. PLoS ONE.

[59] Hausmann R., Hidalgo C.A., Stock D.P., Yildirim M.A. (2014). Implied Comparative Advantage.

